# Plasmid-Mediated Stabilization of Prophages

**DOI:** 10.1128/msphere.00930-21

**Published:** 2022-03-21

**Authors:** Matthew J. Tuttle, Frank S. May, Jonelle T. R. Basso, Eric R. Gann, Julie Xu, Alison Buchan

**Affiliations:** a Department of Microbiology, University of Tennessee, Knoxville, Tennessee, USA; b Department of Biology, Illinois Wesleyan University, Bloomington, Illinois, USA; University of Wisconsin-Madison

**Keywords:** temperate phages, plasmids, mobile genetic elements, spontaneous prophage induction, lysogenic-lytic switch, marine

## Abstract

Mobile genetic elements (MGEs) drive bacterial evolution, alter gene availability within microbial communities, and facilitate adaptation to ecological niches. In natural systems, bacteria simultaneously possess or encounter multiple MGEs, yet their combined influences on microbial communities are poorly understood. Here, we investigate interactions among MGEs in the marine bacterium Sulfitobacter pontiacus. Two related strains, CB-D and CB-A, each harbor a single prophage. These prophages share high sequence identity with one another and an integration site within the host genome, yet these strains exhibit differences in “spontaneous” prophage induction (SPI) and consequent fitness. To better understand mechanisms underlying variation in SPI between these lysogens, we closed their genomes, which revealed that in addition to harboring different prophage genotypes, CB-A lacks two of the four large, low-copy-number plasmids possessed by CB-D. To assess the relative roles of plasmid content versus prophage genotype on host physiology, a panel of derivative strains varying in MGE content were generated. Characterization of these derivatives revealed a robust link between plasmid content and SPI, regardless of prophage genotype. Strains possessing all four plasmids had undetectable phage in cell-free lysates, while strains lacking either one plasmid (pSpoCB-1) or a combination of two plasmids (pSpoCB-2 and pSpoCB-4) produced high (>10^5^ PFU/mL) phage titers. Homologous plasmid sequences were identified in related bacteria, and plasmid and phage genes were found to be widespread in *Tara* Oceans metagenomic data sets. This suggests that plasmid-dependent stabilization of prophages may be commonplace throughout the oceans.

**IMPORTANCE** The consequences of prophage induction on the physiology of microbial populations are varied and include enhanced biofilm formation, conferral of virulence, and increased opportunity for horizontal gene transfer. These traits lead to competitive advantages for lysogenized bacteria and influence bacterial lifestyles in a variety of niches. However, biological controls of “spontaneous” prophage induction, the initiation of phage replication and phage-mediated cell lysis without an overt stressor, are not well understood. In this study, we observed a novel interaction between plasmids and prophages in the marine bacterium Sulfitobacter pontiacus. We found that loss of one or more distinct plasmids—which we show carry genes ubiquitous in the world’s oceans—resulted in a marked increase in prophage induction within lysogenized strains. These results demonstrate cross talk between different mobile genetic elements and have implications for our understanding of the lysogenic-lytic switches of prophages found not only in marine environments, but throughout all ecosystems.

## INTRODUCTION

Bacteria harbor a diverse array of mobile genetic elements (MGEs), including plasmids, phages, gene transfer agents (GTAs), transposons, and genomic islands (1, 2). As primary vectors of horizontal gene transfer (HGT), MGEs drive bacterial evolution and alter genetic diversity within microbial communities (3). In diverse environments, bacteria are likely to possess or encounter several MGEs simultaneously, and some studies have shown that interactions among MGEs alter the transmission and maintenance of plasmids (4–8), host evolutionary responses to individual MGEs (9), and host fitness (10). Most MGE interactions observed to date appear costly to host fitness. Plasmid-mediated activation of the host SOS response can trigger induction of phages and genomic islands (11). In turn, genomic islands have been shown to interfere with plasmid partitioning (6). Additionally, alterations in gene expression profiles of prophage and genomic island-encoded proteins compromising bacterial fitness have resulted from plasmid acquisition (12). Conversely, potential positive fitness effects resulting from MGE interactions are less clear, and a greater understanding of the combined influence of MGE interactions on host evolution and fitness is needed.

MGEs are widespread among marine members of the *Rhodobacteraceae* (roseobacters), where they have been implicated as drivers of adaptation to various ocean niches, and contribute to the ecological success of lineage members (13). Roseobacter plasmids encode a variety of functional traits, including aerobic anoxygenic photosynthesis, flagellar motility, biofilm formation, oxidative stress responses, and pathogenesis of algae. These plasmid-encoded functions are predicted to be important for roseobacter interactions with other organisms and their environment (14–20). Individual roseobacter strains can harbor up to a dozen low-copy-number plasmids with a significant size range (e.g., 5 to 255 kb in Marinovum algicola), amounting to up to a third of their genomic content (18, 21). Many of these plasmids are maintained using RepABC replication modules, which are thus far exclusive to the *Alphaproteobacteria* (22). Upward of 10 distinct roseobacter RepABC plasmid incompatibility groups have been described (18, 23). Conjugal transfer of RepABC plasmids has been demonstrated across roseobacter genera (13, 24), and a roseobacter-like RepABC plasmid has been demonstrated to be stably maintained in the distal alphaproteobacterium Agrobacterium tumefaciens (25).

In addition to harboring an impressive number of plasmids, roseobacters are susceptible to infection by genetically diverse phages, including temperate phages. At least 15 isolated roseophages encode integrase genes, suggestive of a temperate lifestyle (26, 27). Moreover, prophage detection within publicly available genomes also reveals a high incidence of lysogeny within roseobacters (estimated at 26 to 80% [28, 29]).

In this study, we focus on roseobacters of the genus *Sulfitobacter*, which are ubiquitous in the world’s oceans and are known to switch between planktonic and surface-associated modes of growth (17, 30, 31). We previously described a roseobacter model system in which two genetically similar temperate phages influence the fitness of their common host, Sulfitobacter pontiacus, in a growth-modality-dependent fashion (32). *S. pontiacus* can be lysogenized by two different prophage genotypes, influencing interstrain competition dynamics. “Spontaneous” prophage induction (SPI), that is, induction of the lytic cycle in the absence of an overt stressor, is hypothesized to play a role in this strain-to-strain competition (32). Among the limited bacterial taxa in which SPI has been studied, it is known to occur at low rates in lysogenized populations (32–37) and can influence bacterial fitness (32, 38). While little is known of the mechanisms by which SPI occurs, stochastic gene expression and induction of the host SOS response are proposed to play a role in induction of some prophages (33–35). There is evidence supporting a role for host stress in prophage induction in our roseobacter system (32), however, the underlying mechanism initiating that response and whether that is the cause or effect of prophage induction has yet to be elucidated. Here, we sought to explore how components of the *S. pontiacus* genome may contribute to SPI by different prophage genotypes. We present evidence for plasmid-dependent stabilization of prophages in the lysogenic state and posit that this type of interaction may be widespread among natural roseobacter populations.

## RESULTS

### Host genome composition and features.

**FIG 1:**
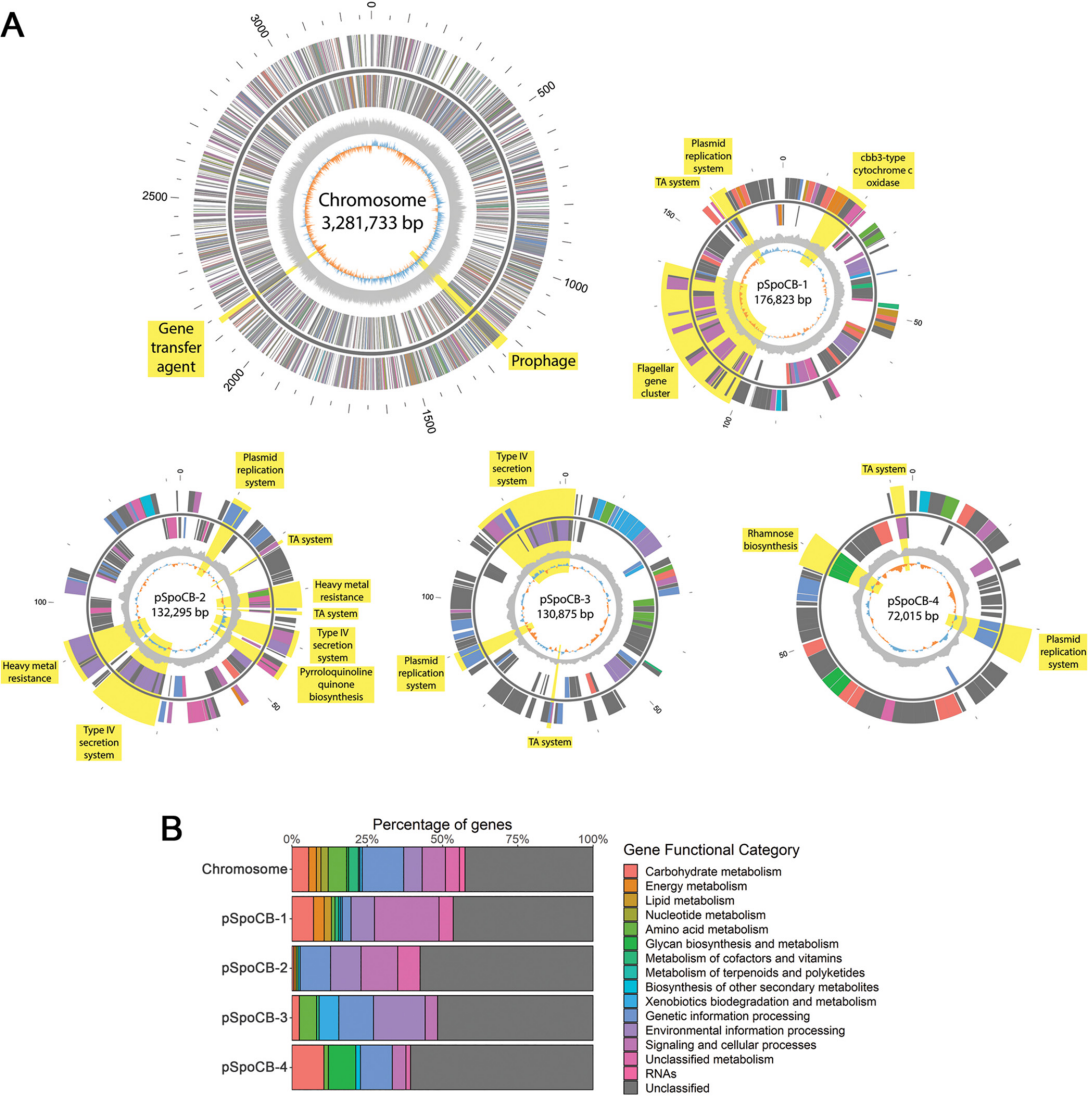
Genomic composition of Sulfitobacter pontiacus CB-D. (A) Circular maps of chromosome and plasmids. Regions of interest are highlighted in yellow. From the outside inward are the following tracks: (1) genomic location in kb, (2) forward strand genes (colored according to broad gene functional categories based on KEGG Orthology), (3) circular chromosome or plasmid DNA (dark gray), (4) reverse-strand genes (same colors as forward-strand genes), (5) GC content (light gray), and (6) GC skew (blue and orange). (B) Percentage of genes on each genomic element belonging to broad gene functional categories based on KEGG Orthology.

To more fully assess the genomic composition of *Sulfitobacter* strain CB-D, long-read whole-genome sequencing was performed using MinION sequencing technology, and reads were coassembled with Illumina reads from Basso et al. (32), resulting in closure of the genome. Phylogenetic analysis of the genome with other related strains revealed that this organism is within the species Sulfitobacter pontiacus (Fig. S1). The closed genome is 3.79 Mb and comprises one circular chromosome (3.28 Mb) and four plasmids (termed pSpoCB-1 through -4), ranging in size from 72 to 177 kb (Table S1 and Fig. 1). With 13.5% of the genome characterized as extrachromosomal, this strain is typical of other strains within the *Sulfitobacter* genus for which complete genomes are available (Table S2). Compared to the original draft genome, the closed genome is 25,951 bp larger, due to the discovery of repeat regions within the genome. These repeat regions include three rRNA operon copies, two elongation factor Tu copies, and duplicate IS*3* and IS*256* family transposon insertion sequences on the chromosome and pSpoCB-4, respectively (Table S1). Roughly half of the genes encoded on the chromosome and each of the plasmids have no known function according to BlastKOALA (Fig. 1B). The previously identified mitomycin-C-inducible prophage harbored by CB-D (φ-D) and a GTA were found to be located on the chromosome, as anticipated (Fig. 1A).

10.1128/msphere.00930-21.1 TABLE S1 Sulfitobacter pontiacus CB-D genome properties Table S1, PDF file, 0.1 MB. Copyright © 2022 Tuttle et al. 2022 Tuttle et al. https://creativecommons.org/licenses/by/4.0/ This content is distributed under the terms of the Creative Commons Attribution 4.0 International license.

10.1128/msphere.00930-21.2 TABLE S2 Properties of complete *Sulfitobacter* genomes Table S2, PDF file, 0.2 MB. Copyright © 2022 Tuttle et al. 2022 Tuttle et al. https://creativecommons.org/licenses/by/4.0/ This content is distributed under the terms of the Creative Commons Attribution 4.0 International license.

10.1128/msphere.00930-21.6 FIG S1 Phylogenetic tree of Sulfitobacter pontiacus CB-D and closely related organisms. This tree was generated by the Type Strain Genome Server (71) and was inferred with FastME version 2.1.6.1 from Genome BLAST Distance Phylogeny (GBDP) distances calculated from genome sequences. The branch lengths are scaled in terms of GBDP distance formula d5. The numbers below branches are GBDP pseudobootstrap support values of >60% from 100 replications, with an average branch support of 64.7%. The tree was rooted at the midpoint. Categorical and genome composition data are represented to the right of each taxon with the following columns from left to right: (i) species cluster, colored according to grouping; (ii) subspecies cluster, colored according to grouping; (iii) percent GC content, colored as a heatmap (white to blue); (iv) delta statistic, colored as a heatmap (white to red); (v) genome size (black), scaled according to size; (vi) protein count (tan), scaled according to count; (vii) indicator of user-supplied strain genome (blue plus); and (viii) indicator of type species (red circle). Download FIG S1, PDF file, 0.09 MB. Copyright © 2022 Tuttle et al. 2022 Tuttle et al. https://creativecommons.org/licenses/by/4.0/ This content is distributed under the terms of the Creative Commons Attribution 4.0 International license.

**FIG 2:**
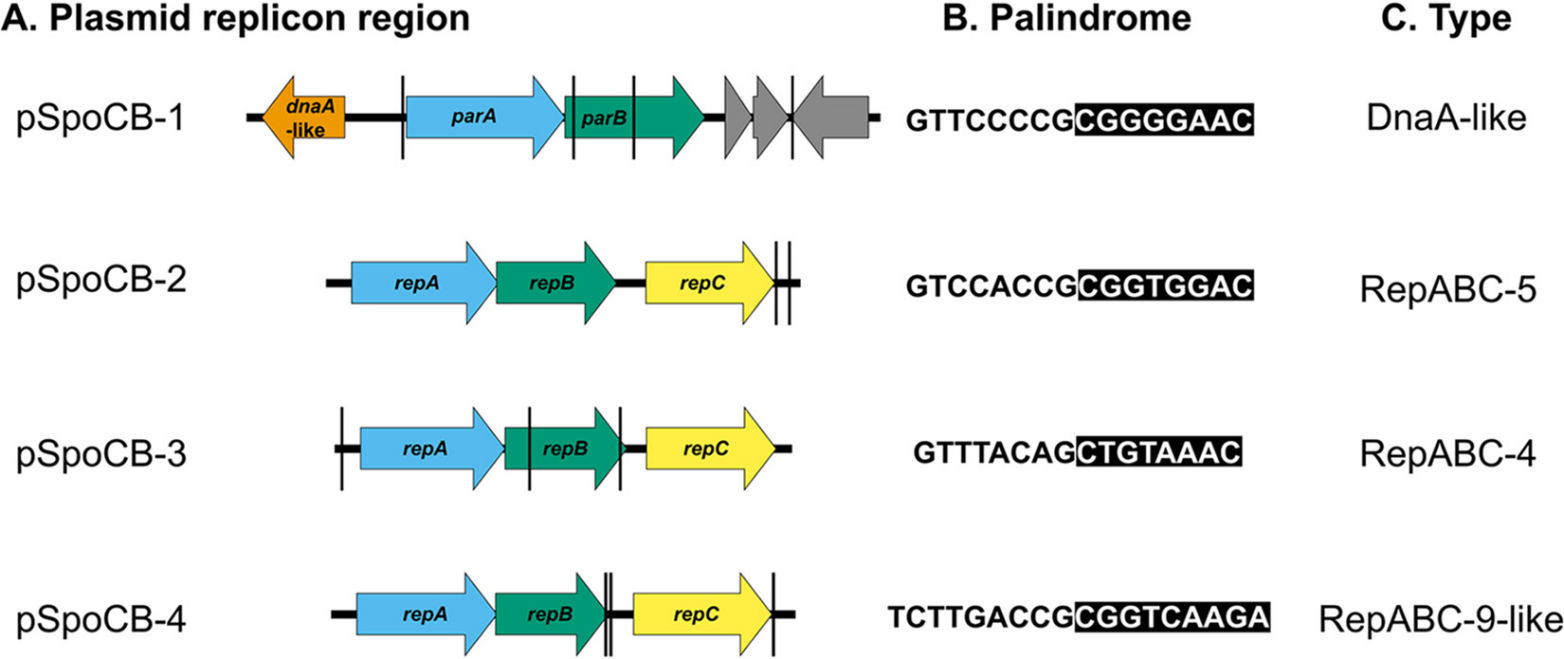
Organization of CB-D plasmid replication modules. (A) Plasmid replicon regions with arrows indicating genes involved in plasmid replication and partitioning. Vertical black lines indicate locations of palindromic incompatibility regions. (B) Identified palindromic sequences unique to each plasmid. (C) Plasmid compatibility group based on genetic organization and palindromic sequences identified (compared to those previously described; 25, 69).

All four CB-D plasmids are low copy number with plasmid-to-chromosome (P-C) ratios ranging from 0.45 to 5.51 as measured by quantitative PCR (qPCR) (Table 1). These results are further supported by the relatively even read mapping coverage of Illumina reads between the chromosome (284) and four plasmids (202 to 253; Table S3). Each of the four plasmids was classified to different plasmid incompatibility groups, with a DnaA-like replicon for pSpoCB-1 and three separate RepABC replicons for the remaining plasmids. Unique palindromic incompatibility (*incβ*) regions were identified for each RepABC plasmid (Fig. 2). For pSpoCB-2 and pSpoCB-3 these regions were identical in sequence and location to replicon types RepABC-5 and RepABC-4, respectively. pSpoCB-4 was identified as RepABC-9-like, as its *incβ* region matched closely to the RepABC-9 consensus palindrome with only one mismatch (Fig. 2). Each plasmid encodes at least one type II toxin-antitoxin system, all of which are unique and predicted to be involved in plasmid maintenance (39). pSpoCB-1 possesses the only flagellar gene cluster in the genome (similar to other roseobacter plasmids [18, 40]), cytochrome *c* oxidase genes, and several genes implicated in cellular stress responses (e.g., beta lactamase, thioredoxin peroxidase, and a cbb3-type cytochrome *c* oxidase). pSpoCB-2 and pSpoCB-3 each encode a unique type IV secretion system, indicating that these plasmids may be conjugative (Fig. S2). pSpoCB-2 also encodes putative heavy metal resistance genes, while pSpoCB-3 encodes genes involved in aromatic carbon catabolism, and pSpoCB-4 encodes a rhamnose biosynthetic operon.

**TABLE 1:** Relative copy number of plasmids as assessed by quantitative PCR

Plasmid	P-C ratio^*a*^ (mean ± SD)	
	CB-D	CB-A^*b*^
pSpoCB-1	2.07 ± 0.64	2.01 ± 0.40
pSpoCB-2	1.42 ± 0.45	ND
pSpoCB-3	5.51 ± 1.86	5.49 ± 1.24
pSpoCB-4	0.45 ± 0.19	ND

*^a^*Ratio of plasmid copies to chromosome copies.

*^b^*ND, not detected (<10^−4^ copies per chromosome copy).

10.1128/msphere.00930-21.3 TABLE S3 Sequencing coverage statistics of CB-D and CB-A Illumina reads (32) mapped to the complete CB-D genome Table S3, PDF file, 0.1 MB. Copyright © 2022 Tuttle et al. 2022 Tuttle et al. https://creativecommons.org/licenses/by/4.0/ This content is distributed under the terms of the Creative Commons Attribution 4.0 International license.

10.1128/msphere.00930-21.7 FIG S2 Alignment of pSpoCB-2 and pSpoCB-3 T4SS operons. ORFs are depicted by light blue arrows with conservation of nucleotides shown on a scale from 0% (yellow) to 100% (blue). Gaps in the alignment are indicated by light gray boxes. Download FIG S2, PDF file, 0.08 MB. Copyright © 2022 Tuttle et al. 2022 Tuttle et al. https://creativecommons.org/licenses/by/4.0/ This content is distributed under the terms of the Creative Commons Attribution 4.0 International license.

### Plasmid content differs between CB-D and CB-A.

*S. pontiacus* CB-A was previously generated via superinfection of CB-D with exogenous phage φ-A (32). When exogenous phages (φ-A or φ-D) are used to superinfect *S. pontiacus* strains of the reciprocal phage genotype, this leads to either a lytic infection or prophage genotype conversion, whereby one phage genotype is replaced by the other. We assessed differences in plasmid content between CB-D and its derivative, CB-A, as Illumina reads from Basso et al. (32) only mapped to the chromosome and pSpoCB-3 (Table S3). PCR amplification of regions specific to each plasmid revealed that CB-A possesses pSpoCB-1 and pSpoCB-3. Like CB-D, these plasmids are present in the strain at similarly low copy numbers, 2.01 for pSpoCB-1 and 5.49 for pSpoCB-3 (Table 1). Flagella present in electron micrographs of CB-D and CB-A indicate that both strains indeed possess pSpoCB-1, as this plasmid encodes the only flagellar gene cluster in the genome (Fig. S3).

10.1128/msphere.00930-21.8 FIG S3 Electron micrographs of parental strains. Images are representative of typical strain morphology for CB-D (left) and CB-A (right) with arrows indicating flagella. Download FIG S3, PDF file, 0.1 MB. Copyright © 2022 Tuttle et al. 2022 Tuttle et al. https://creativecommons.org/licenses/by/4.0/ This content is distributed under the terms of the Creative Commons Attribution 4.0 International license.

### Derivative strains reveal interactions between phages and plasmids.

The prophages within CB-D and CB-A (here termed prophage-D and prophage-A, respectively) share a integration site within *S. pontiacus* and are incompatible with one another within a single genome (32). Given the differences in plasmid content between CB-D and CB-A (Table 1) and previously measured phenotypic differences between these strains (32), we sought to derive new strains via superinfection to swap prophage genotypes in different host genetic backgrounds (i.e., host plasmid content; Fig. S4). These derivative strains were used to assess if plasmid loss occurs concurrently to prophage genotype switching and allowed for assessment of physiology compared to the parental strains. Following superinfections, colonies were serially streaked for isolation and screened for the presence of different phage genotypes using phage-specific primers (Fig. S4). From superinfection of CB-D with φ-A, 8 new derivative strains harboring the prophage-A genotype (CB-A1-1 to -8) were identified. From the converse superinfection of CB-A with φ-D, 4 additional derivative strains containing the prophage-D genotype (CB-D1-1 to -4) were isolated. Chromosomal integration of phages was confirmed via PCR amplification across the junctions of phage and host chromosome DNA at the common integration site.

10.1128/msphere.00930-21.9 FIG S4 Generation of derivative strains via prophage genotype swapping and incidence of lysogenic conversion. (A and B) These diagrams outline culturing and screening of colonies in the generation of derivative CB-A (A) and CB-D (B) strains via superinfections. Tables indicate PCR screening results of picked colonies 8 and 24 h post infection (hpi), with strains that underwent lysogenic conversion shown in bold. (C and D) Diagram of plasmid and prophage contents for prophage-D-containing derivatives of CB-A (C) and prophage-A-containing derivatives of CB-D (D). (E) Phage dilution assay of parental and derivative strains. Free phages were harvested from cultures after 24 h growth in broth culture in the absence of induction. For each strain, biological and technical triplicates are shown. Phage dilutions were inoculated onto host organisms susceptible to lysis by the respective phage types (CB-A for φ-D and CB-D for φ-A). Controls represent phage-free medium inoculated onto hosts (left, CB-D; right, CB-A). Download FIG S4, PDF file, 0.3 MB. Copyright © 2022 Tuttle et al. 2022 Tuttle et al. https://creativecommons.org/licenses/by/4.0/ This content is distributed under the terms of the Creative Commons Attribution 4.0 International license.

**FIG 3:**
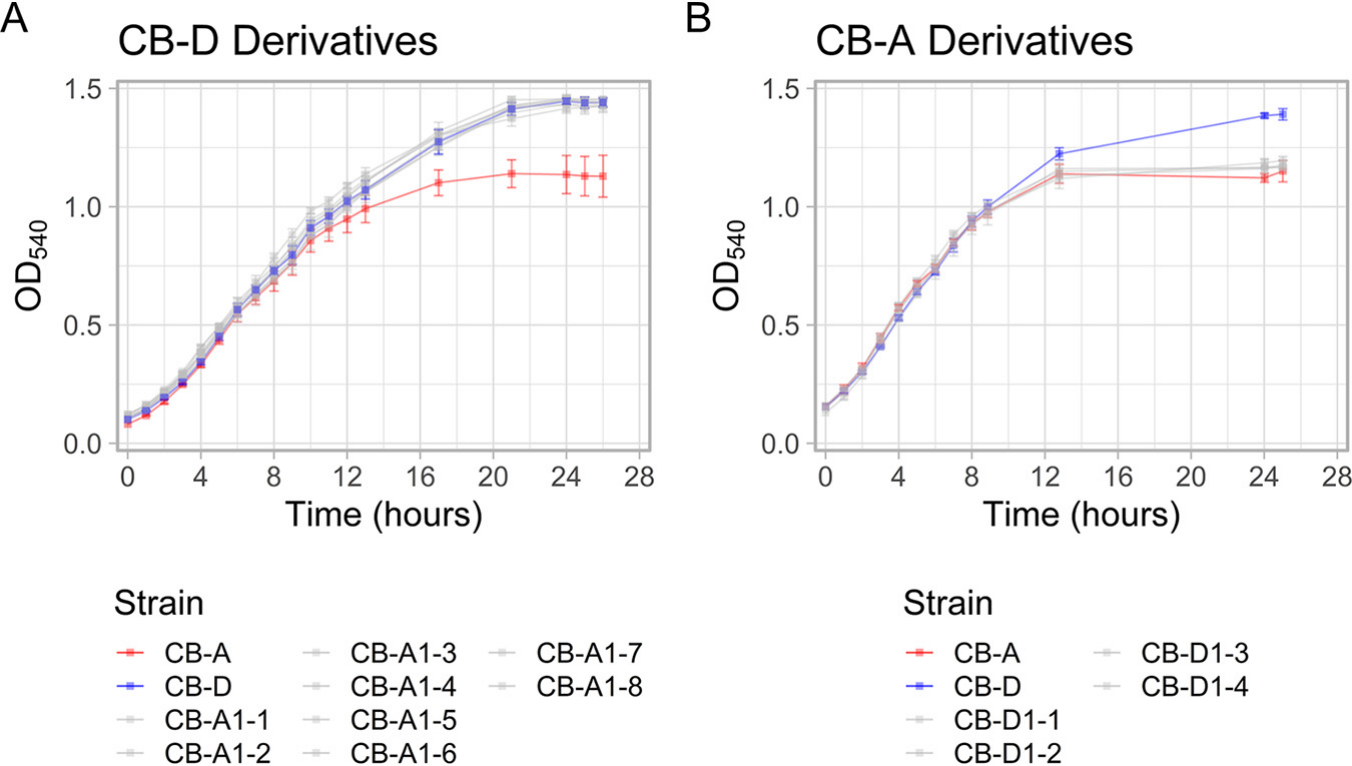
Growth dynamics of parental and derivative *S. pontiacus* strains. (A and B) Prophage-A-lysogenized derivative strains (A) and prophage-D-lysogenized derivative strains (B) compared to parental strains CB-D (blue) and CB-A (red) in liquid culture. Points denote the mean of biological triplicates, and error bars indicate the standard deviation from the mean at each time point.

**FIG 4:**
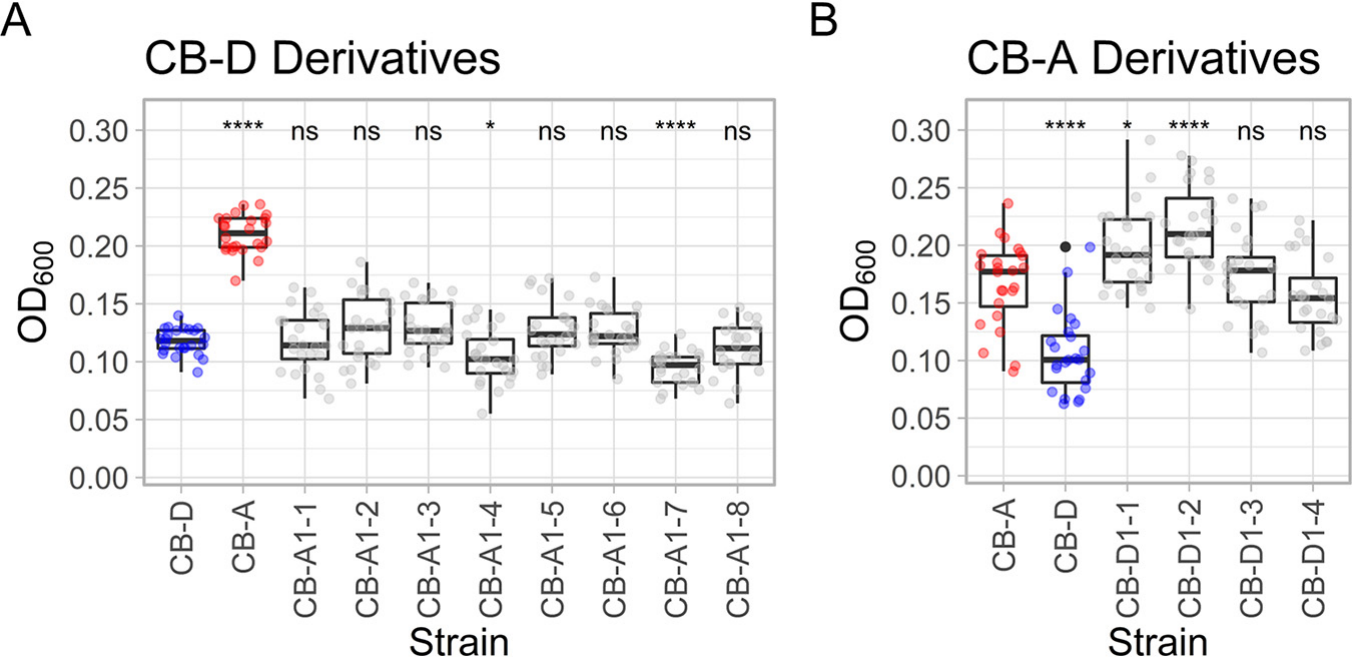
Relative biofilm formation of parental and derivative *S. pontiacus* strains. (A and B) Crystal violet biofilm assays of prophage-A-lysogenized derivative strains (A) and prophage-D-lysogenized derivative strains (B) compared to parental strains CB-D (blue) and CB-A (red). Plots depict the median (bold line), 25th and 75th percentiles (box), 1.5 times the interquartile ranges (whiskers), and outliers (black dots) with all replicates overlaid (transparent circles). For each panel, pairwise Wilcoxon tests were used to determine significant differences between derivatives and their parental strain (CB-D for panel A; CB-A for panel B). Significant differences are denoted by asterisks (ns, not significant, *P* > 0.05; *, *P* ≤ 0.05; **, *P* ≤ 0.01; ***, *P* ≤ 0.001; ****, *P* ≤ 0.0001).

**FIG 5:**
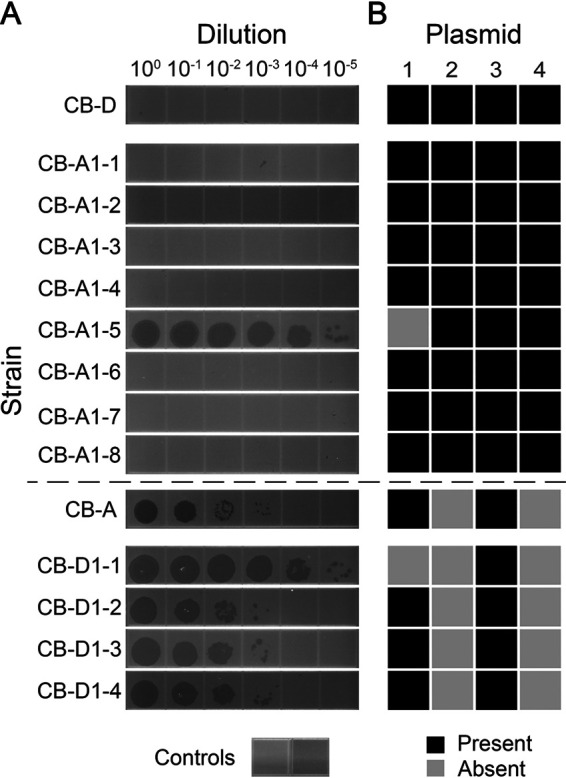
*S. pontiacus* strains lacking plasmids have high free-phage titers in the absence of induction. (A) Phage dilution assay showing titers of phage from cultures after 24 h growth in SMM broth without exogenous induction. Phage serial dilutions (10 μL) were inoculated onto host organisms susceptible to lysis by the respective phage types (CB-A for φ-D and CB-D for φ-A). Controls represent phage-free medium inoculated onto hosts (left, CB-D; right, CB-A). See Fig. S4E for images of all replicates. (B) Presence of plasmids pSpoCB-1 through pSpoCB-4 within strains. Plasmid presence was determined via amplification of genomic DNA from strains using at least three primer sets unique to each plasmid.

Growth dynamics of all derivative strains mimicked that of the parent from which they were derived (Fig. 3). The same trend was seen with relative biofilm formation as measured by a standard crystal violet biofilm assay (Fig. 4). Spontaneous prophage induction differed among strains, with some exhibiting no detectable level of phage production (<10^−1^ PFU/mL), while others exhibited high phage titers (>10^5^ PFU/mL). Most notably, a robust link between plasmid content and phage titers in cell-free lysate (indicative of SPI) was observed (Fig. 5). Regardless of prophage genotype, lysogens possessing all four plasmids had undetectable levels of phage titers. The only new prophage-A lysogen with detectable phage production was CB-A1-5 (>10^7^ PFU/mL), which lacks pSpoCB-1. Similarly, all newly generated prophage-D lysogens, lacking pSpoCB-2 and pSpoCB-4, had phage titers consistent with their prophage-A containing parent (>10^5^ PFU/mL). One of these strains (CB-D1-1) also lacks pSpoCB-1 and exhibited higher phage titers (>10^7^ PFU/mL; Fig. 5A and Fig. S4). pSpoCB-1 was the only plasmid lost during generation of the new derivative strains (Fig. 5B).

### Plasmid pSpoCB-1 is not stably maintained in the host, regardless of prophage genotype.

**FIG 6:**
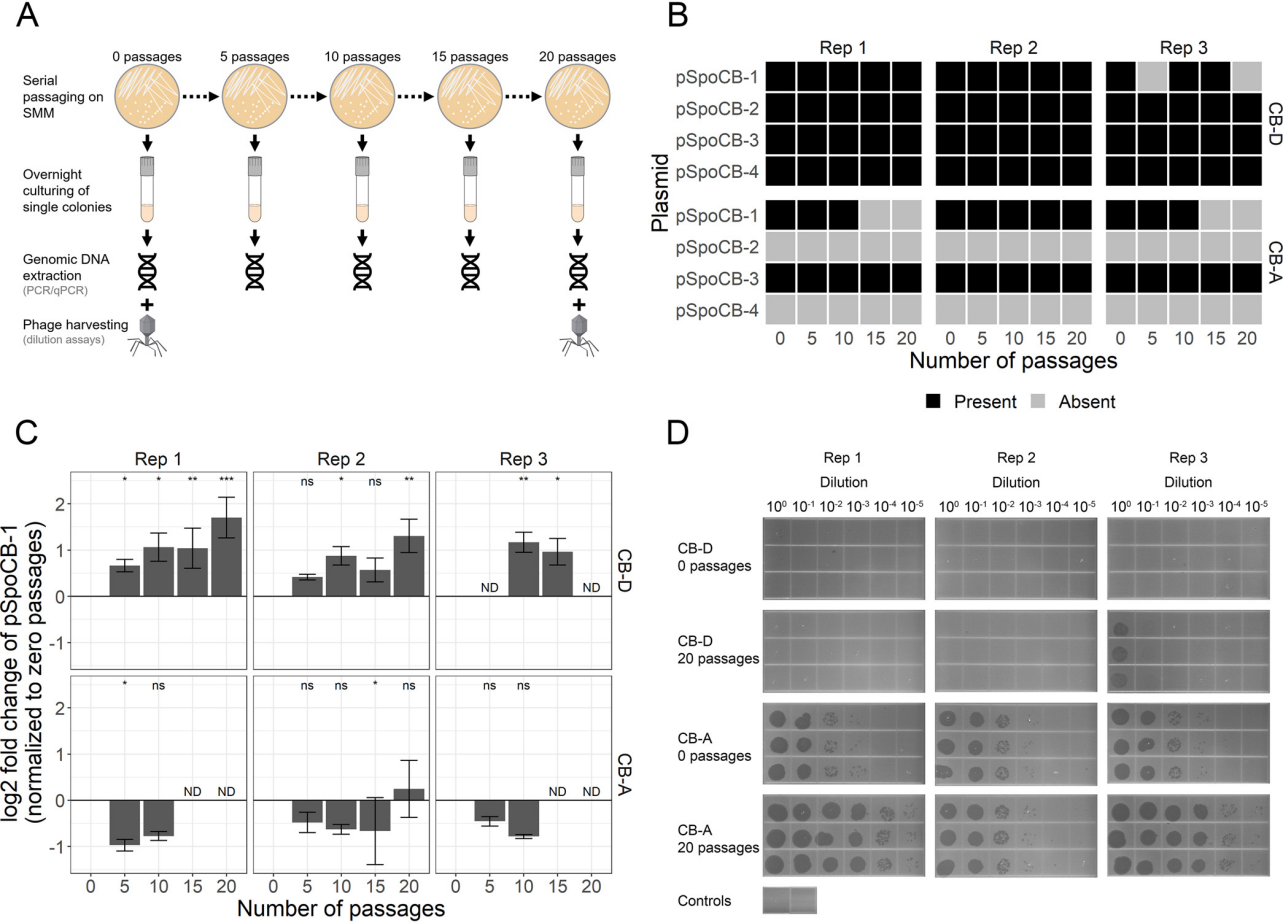
Plasmids appear stable after multiple passages on agar medium, except for pSpoCB-1. (A) Diagram of serial passaging and screening of colonies. (B) The parental strains CB-D (top) and CB-A (bottom) were serially passaged in triplicate (Rep 1 to 3) on agar plates for a total of 20 passages. Every 5 passages, the presence of individual plasmids was assessed via PCR amplification of genomic DNA with at least two primer sets that amplify unique regions of their respective plasmid. (C) Fold change of pSpoCB-1 abundance with serial passaging on agar as determined by qPCR. Data were normalized to zero passages on agar and represent mean values of pSpoCB-1-specific qPCRs relative to single-copy chromosomal gene qPCRs (*map* and *alaS*). ND (no detection), indicating samples with plasmid copies below the assay limit of detection. Significant differences (Student’s *t* tests) in Δ*C_T_* values compared to zero passages for each replicate are denoted by asterisks (ns, not significant, *P* > 0.05; *, *P* ≤ 0.05; **, *P* ≤ 0.01; ***, *P* ≤ 0.001). (D) Phage dilution assay showing titers of free phage from cultures after 24 h growth in broth culture in the absence of induction. Phage dilutions were inoculated onto host organisms susceptible to lysis by the respective phage types (CB-A for φ-D and CB-D for φ-A). For each strain, biological triplicates (columns) and technical triplicates (rows) are shown. Controls represent phage-free medium inoculated onto hosts (left, CB-D; right, CB-A).

As pSpoCB-1 was lost among a subset of the derivative strains during their generation, we next assessed the stability of all plasmids within the parental strains CB-D and CB-A by serial passage on agar (20 times). Every 5 passages, individual colonies were PCR screened for the presence of plasmids (Fig. 6A). Three plasmids (pSpoCB-2, -3, and -4) were stably maintained over time, whereas pSpoCB-1 was lost from one CB-D replicate and two CB-A replicates (Fig. 6B). This result was further confirmed using qPCR (Fig. 6C). Consistent with observations made in strains with various prophage/plasmid combinations derived from superinfection experiments (Fig. 5), strains lacking pSpoCB-1 showed measurable (CB-D derivatives) or elevated (CB-A derivatives) phage titers relative to their parent strain (Fig. 6D).

### Plasmids share homology with other sequenced roseobacter plasmids.

**FIG 7:**
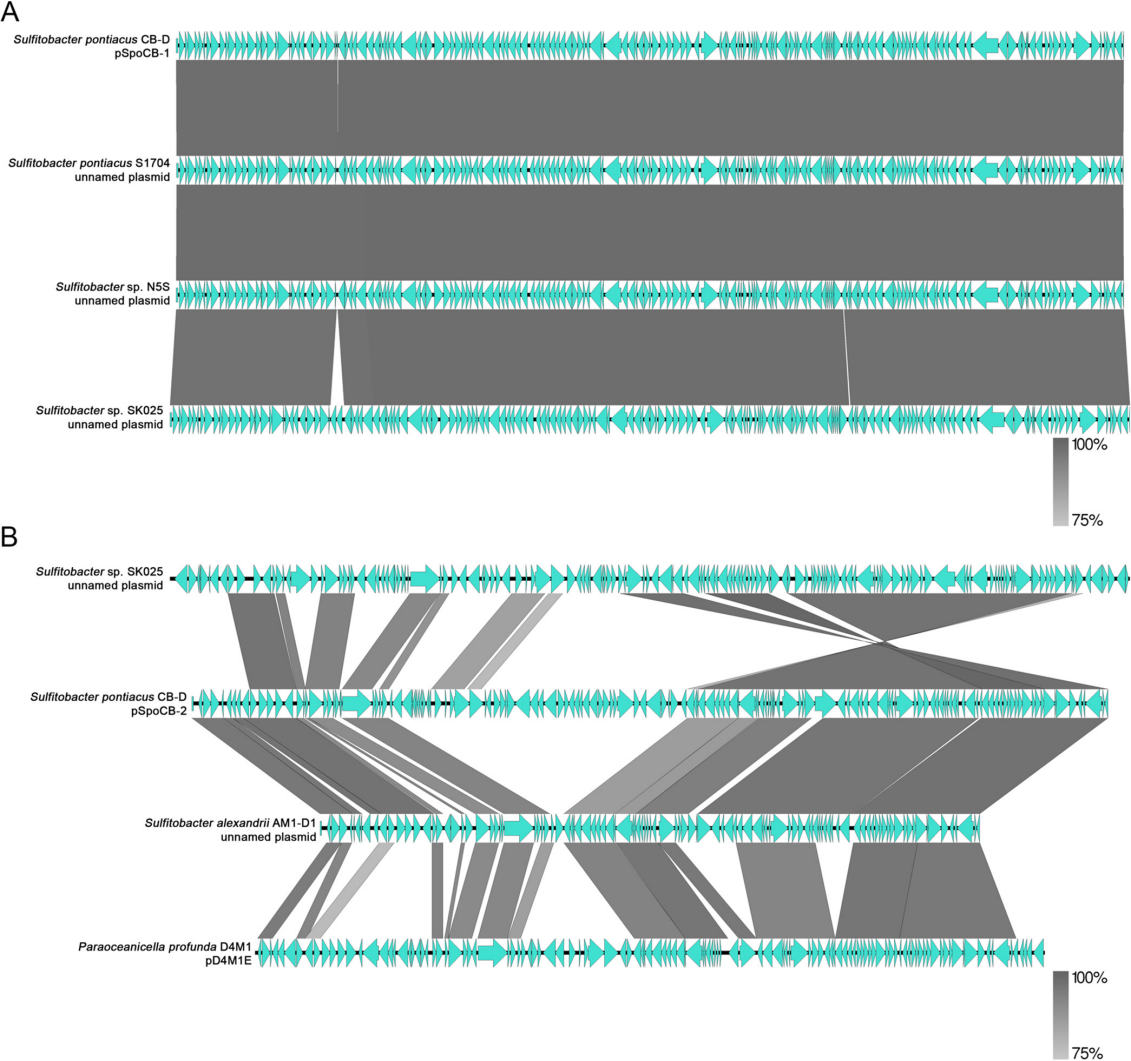
pSpoCB-1 and pSpoCB-2 share a high degree of synteny with other sequenced *Sulfitobacter* plasmids. (A) pSpoCB-1 and (B) pSpoCB-2 compared to other sequenced roseobacter plasmids. Blue arrows represent ORFs for individual plasmids. Gray bars indicate long-range homology between plasmids with ≥75% nucleotide sequence similarity. Plasmid sequences were downloaded from NCBI. The accession numbers for panel A are CP072614 (CB-D), CP049345 (S1704), JACIFR010000004 (N5S), CP025810 (SK025). The accession numbers for panel B are CP025811 (SK025), CP072615 (CB-D), CP018078 (AM1-D1), CP040823 (D4M1).

Plasmid sequences exhibiting long-range synteny and nucleotide homology with pSpoCB-1 and pSpoCB-2 were identified in other sequenced roseobacter genomes. For pSpoCB-1, nearly identical plasmids were found within the genomes of *Sulfitobacter* sp. strains S1704, N5S, and SK025, as they shared 97.64%, 97.76%, and 95.89% nucleotide identity to pSpoCB-1, respectively (Fig. 7A). pSpoCB-2-like plasmids were found in the genomes of *Sulfitobacter* sp. strain SK025, Sulfitobacter alexandrii AM1-D1, and Paraoceanicella profunda D4M1. These plasmids shared 45.80%, 59.87%, and 49.41% nucleotide identity to pSpoCB-2, respectively (Fig. 7B). In contrast, no pSpoCB-3- or pSpoCB-4-like plasmids were identified.

### Sulfitobacter plasmid genes are widely distributed in the oceans.

**FIG 8:**
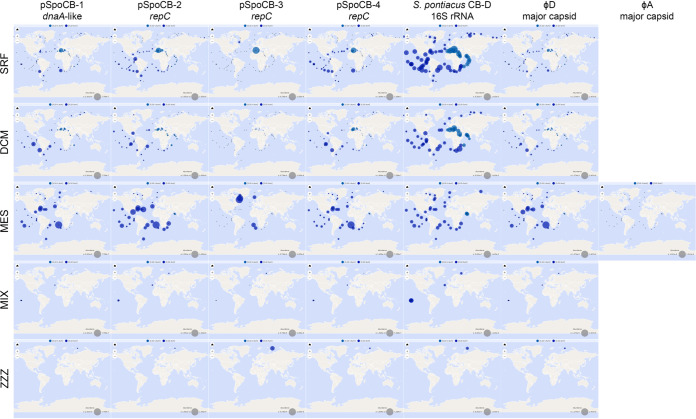
Biogeographical distribution and relative abundance of host and phage genes in the oceans. Each column represents a single gene located on either a plasmid or the chromosome or within phage genomes. Each row represents a different sampling depth from the *Tara* Oceans Expedition (SRF, surface ocean; DCM, deep chlorophyll maximum; MES, mesopelagic; MIX, marine epipelagic mixed layer; ZZZ, marine water layer). All *Tara* Oceans sampling stations and depths are indicated with an X. Blue circles represent the abundance of BLAST hits of genes to the OM-RGCv2 database with a cutoff threshold of 10^−10^. Abundance is normalized as a percentage of the total reads within that sample. Maps are omitted for depths at which no BLAST hits to genes were detected (i.e., for the φ-A major capsid protein). Individual maps were generated by the Ocean Gene Atlas online server (77).

To assess the biogeographical distribution of plasmids in the ocean that are like those harbored by *S. pontiacus* CB-D, we performed homology searches of plasmid replicon genes against the *Tara* Oceans Microbiome Reference Gene Catalog database. In the prokaryotic-sized (0.22 to 1.6 and 0.22 to 3 μm) seawater fractions collected by the *Tara* Oceans expedition, being composed of planktonic cells, plasmid replicon genes for all four plasmids were found to be widespread (Fig. 8). pSpoCB-1, pSpoCB-2, and pSpoCB-4 replicon genes were found at most stations and across all regions sampled at a variety of depths. The *repC* gene of pSpoCB-3 was also found across different depths but was limited to Arctic Ocean, Atlantic Ocean, and Mediterranean Sea samples. The highest relative abundance of all plasmid replicon genes was in mesopelagic samples. This corresponded with the highest abundance of the φ-D major capsid protein homologs, and the mesopelagic was the only depth at which major capsid protein homologs of φ-A were found. These contrast with *Sulfitobacter* 16S rRNA genes, as they were present across all oceans and depths but had the highest relative abundance in surface ocean samples. To determine if plasmid genes other than the plasmid replicons are present together in the oceans, we performed additional homology searches, this time using whole-plasmid sequences against the *Tara* Oceans prokaryotic assemblies (41). From these searches, metagenome sequence hits up to 33,858 bp long, covering up to 26% of the plasmid queried, were identified (Fig. S5A). These hits were not isolated to single regions of these plasmids. The top 30 BLAST hits for all plasmids came from a variety of sampling stations and depths, with most coming from stations located in the southern Atlantic Ocean (Fig. S5B).

10.1128/msphere.00930-21.10 FIG S5 Top BLAST hits of whole-plasmid sequences searched against the *Tara* Oceans microbiome prokaryotic assemblies. (A) Plots depicting the locations of the top 30 BLAST hits for each plasmid. From the outside inward are the following tracks: (1) nucleotide location in kb, (2) plasmid sequence (teal) and BLAST hits (blue), and (3) multicolored chords depicting regions of homology between plasmid sequence and BLAST hits. (B) Treemaps depicting the distribution of *Tara* Oceans sampling locations and depths for the top 30 BLAST hits to each plasmid. Sampling stations in the southern Atlantic Ocean are highlighted in blue. Depth abbreviations are as follows: SRF, surface water; DCM, deep chlorophyll maxima; MES, mesopelagic. Download FIG S5, PDF file, 0.2 MB. Copyright © 2022 Tuttle et al. 2022 Tuttle et al. https://creativecommons.org/licenses/by/4.0/ This content is distributed under the terms of the Creative Commons Attribution 4.0 International license.

## DISCUSSION

Through their transfer and interactions with hosts, MGEs exert considerable influence over the evolution of bacterial genomes and overall functions of bacterial communities (3). However, despite their prevalence among microbial genomes, and the diversity of MGEs a single host can possess, few studies explicitly examine interactions among MGEs. This is especially true of interactions between different types of MGEs (e.g., plasmids and phages [4]). Here, we examined interactions between two temperate phages and naturally occurring plasmids within the marine bacterium *S. pontiacus* CB-D. We show a stark relationship between the absence of certain plasmids and prophage induction. This finding is similar to a plasmid-phage pair in Enterococcus faecalis, where an increased basal level of prophage production was briefly noted in a plasmid-cured strain but not further characterized (42). In our system, this relationship is not limited to one plasmid-prophage pair; rather, lysogens containing either prophage-A or prophage-D exhibited high rates of SPI, as measured by phage titers, within a variety of host strain backgrounds. This includes strains lacking (i) pSpoCB-1 alone, (ii) pSpoCB-2 and pSpoCB-4 together, or (iii) pSpoCB-1, pSpoCB-2, and pSpoCB- 4 (Fig. 3). Furthermore, given the difference in apparent magnitude of SPI between strains that contain prophage-A (high titers; Fig. 5A) or prophage-D (low titers; Fig. 6D) with the same plasmid content (i.e., lacking only pSpoCB-1), there may be a genotype specificity in this plasmid-prophage interaction related to the divergent transcriptional regulators found in these phages (32). Collectively, these results suggest either multiple mechanisms by which individual (or combinations of) plasmids influence SPI or, alternatively, a similar mechanism that exists across these genetically distinct plasmids.

The differences in SPI found among the strains presented here are apt to influence the dynamics of cell-to-cell competitions and development of microbial community structures. In other organisms, SPI has been shown to increase the fitness of both lysogens and phages (38). Increased competitiveness of lysogens using phages as weapons in competition is a proposed benefit to harboring prophages (43), and predation of neighboring cells can enhance HGT by yielding extracellular DNA for natural transformation (44). We previously showed increased fitness within strains harboring prophage-A or -D, as these prophages form coalitions with their host, leading to niche-specific fitness effects (32). Our results now reveal that plasmids also appear to play a role in these coalitions by impacting SPI.

While numerous factors have been previously shown to lead to the induction of prophages within bacteria (45), the mechanisms that dictate this lysogenic-lytic switch remain poorly understood for most phages. Escherichia coli phage lambda and other lambdoid phages are a notable exception (46). These phages are induced by activation of the host SOS response, initiated by DNA damage within the cell (47). Production of RecA leads to cleavage of the phage-encoded transcriptional repressor that prevents expression of lytic phage replication genes (46). We propose that a similar mechanism exists for φ-A and φ-D, as these phages are mitomycin C-inducible, and SOS gene activation occurs within superinfected populations that are actively producing phages (32). This lysogenic-lytic switch then modulates the degree of SPI in the *S. pontiacus* strains observed here. As no obvious phage defense mechanisms exist on these plasmids, SPI and the resulting phage-mediated cell lysis is likely driven by this genetic switch rather than halted phage production by a defense system at a later stage of replication, as has been seen with some phages that infect cyanobacteria (48).

Stochastic expression of the SOS response has been proposed as a mechanism by which SPI occurs for some phages (33–35). In Pseudomonas fluorescens, fitness costs associated with plasmid carriage have been shown to be linked to the SOS response, the result of plasmid-induced gene expression (49). *S. pontiacus* plasmids could modulate SOS activation by altering the host cell state, in particular, oxidative stress levels. Plasmids of other roseobacters have been shown to play a role in oxidative stress responses (15), and there are several plasmid-encoded stress response genes within *S. pontiacus* CB-D. These include a thioredoxin peroxidase and a cbb3-type cytochrome *c* oxidase on pSpoCB-1. Cbb3-type oxidases are often expressed in low-oxygen environments as a terminal oxidase, but have also been shown to play a protective role against reactive oxygen species (50). pSpoCB-2 encodes a pyrroloquinoline quinone biosynthetic pathway, a cofactor with known oxygen scavenging properties (51), additional cytochrome *c* oxidase genes, a suite of heavy metal resistance genes, including those for Hg and Cu/Ag, and several copper oxidases. Lastly, the rhamnose biosynthetic operon encoded on pSpoCB-4 may also be involved, as disruption of this pathway in Streptococcus mutans was recently seen to increase susceptibility to oxidative stress (52). In the absence of one, or more, of these genes, higher basal levels of oxidative stress may occur, leading to higher rates of observed SPI. Additionally, host stress and activation of the SOS response has been demonstrated to increase rates of HGT, principally by integrative and conjugative elements as well as gene transfer agents (GTAs) (53). Thus, we might expect increased rates of HGT to occur with these *S. pontiacus* strains as well, mediated by (i) increased conjugation or (ii) transduction by the resident prophage.

Loss of pSpoCB-1 with sustained passaging under laboratory conditions was observed. However, this lack of stability was not observed with either pSpoCB-2 or pSpoCB-4. Instead, their initial loss within CB-A may have been the result of the strain’s generation via superinfection affecting plasmid replication or partitioning. In conditions under which plasmids confer no benefits, phages have been shown to accelerate plasmid loss (5). Lytic infection of an archaeon, Acidianus hospitalis W1, by the lipothrixvirus AFV1, was also shown to prevent replication of its plasmid, pHA1, leading to its loss (54). A similar mechanism of plasmid loss might occur for some *S. pontiacus* plasmids, although further investigation is needed to support this hypothesis.

Mechanisms by which phages may influence maintenance of plasmids are largely unknown. Harboring plasmids comes at a fitness cost to host cells (10), which may be attributed to genetic conflicts due to specific deleterious interactions between plasmid and chromosomal genes (49). For vertical transmission to be maintained, plasmids often employ a variety of maintenance strategies in addition to their controlled-replication systems. Known maintenance strategies include multimer resolution systems, postsegregational killing systems, most commonly, toxin-antitoxin addiction modules, and partitioning systems (55). The *S. pontiacus* plasmids pSpoCB-1 through -4 each harbor partitioning systems and at least one toxin-antitoxin (TA) system. The TA systems likely trigger postsegregational killing during cell division when there is improper distribution of plasmids to daughter cells (39). Continuous production of unstable antitoxins is required to counteract more stable cognate toxins, and loss of a plasmid, or the antitoxin gene itself, results in toxin activation. Here, we suggest that phage-mediated postsegregational killing may represent an additional novel mechanism of plasmid maintenance akin to toxin-antitoxin systems within *S. pontiacus*, at least for plasmids pSpoCB-2 and pSpoCB-4. Upon plasmid loss, activation of the lysogenic-lytic switch of prophages would act as an emergency stop on ineffective inheritance of plasmids, thereby leading to greater survival of plasmid-harboring cells.

*S. pontiacus* strains lacking both pSpoCB-2 and pSpoCB-4 form more robust biofilms relative to strains that possess these plasmids. pSpoCB-4 encodes a rhamnose biosynthetic operon, a feature of known “biofilm plasmids” among roseobacters (17). However, SPI is also expected to promote biofilm matrix formation. Prophage induction within biofilms contributes to biofilm development and dispersal, depending on environmental conditions and the individual host strains and prophages involved (56). In some strains, SPI-promoted biofilm development has been attributed to the release of extracellular DNA (57–59), a critical biofilm matrix component (60). As cells within biofilms are in close proximity and diffusion is limited, it is expected that interactions between phages and their hosts are enhanced within biofilms (61). Nutrient and metabolite gradients (e.g., oxygen) within a biofilm matrix could also influence rates of SPI. Further work examining these interactions is therefore needed to improve our understanding of the impact phages have on biofilm physiology and function in natural environments.

Given the abundance and ubiquity of *S. pontiacus* plasmid genes in the *Tara* Oceans data sets, we predict that the plasmid-phage relationship identified here may be prevalent among natural roseobacter populations. Whether this relationship extends to other bacterial taxa remains an open question. Future studies probing the molecular mechanisms underlying this interaction should lead to a better understanding of how genomic content, host physiological state, and environmental conditions together trigger and modulate rates of induction.

## MATERIALS AND METHODS

### Growth conditions and phage isolation.

The strains and phages used in this study are listed in Table S4. All strains were routinely cultured in 10-mL volumes at 25°C in the dark with 200 rpm agitation in standard marine medium (SMM), as previously described (32). Phages were routinely harvested from either uninduced or mitomycin C-induced cultures via centrifugation for 10 min at 5,000 rpm followed by 0.22-μm filtration of the supernatant to obtain the free phage fraction. Prophages were induced via mitomycin C (0.5 μg/mL) addition to exponential-phase cultures (optical density at 540 nm [OD_540_], 0.17; ∼10^7^ CFU/mL) followed by overnight incubation prior to harvesting.

10.1128/msphere.00930-21.4 TABLE S4 List of strains and bacteriophages used in this study Table S4, PDF file, 0.1 MB. Copyright © 2022 Tuttle et al. 2022 Tuttle et al. https://creativecommons.org/licenses/by/4.0/ This content is distributed under the terms of the Creative Commons Attribution 4.0 International license.

### Genome resequencing, annotation, and analysis.

A 12-contig draft genome sequence of Sulfitobacter pontiacus CB-D (formerly named *Sulfitobacter* sp. CB2047) was previously generated using the Illumina sequencing platform (accession number JPOY01000000 [62]). We closed this genome using MinION sequencing. For genomic DNA extraction, overnight cultures of CB-D were grown in SMM, and cells were concentrated via centrifugation for 5 min at 10,000 rpm. Cell pellets were resuspended in 567 μL of 10 mM Tris (pH 8.0), 30 μL of 10% (wt/vol) SDS, and 3 μL of 20 mg/mL proteinase K and incubated for 2 h at 55°C. Two rounds of phenol extraction were performed where 800 μL of saturated phenol with 8-hydroxyquinolone (pH 7.8 to 8.2) was added, samples were spun for 5 min at 10,000 rpm, and the aqueous phase was transferred to a new tube. This was followed by one round of extraction using 800 μL of phenol:chloroform:isoamyl alcohol (25:24:1). To the aqueous phase, 50 μL of 7.5 M ammonium acetate and ∼1.5 mL of absolute ethanol were added prior to incubation at –20°C overnight. Samples were then spun for 10 min at 10,000 rpm at 4°C, and pellets were washed with 0.5 mL of 70% ethanol and spun again. After the supernatant was poured off, DNA pellets were air dried and resuspended in 100 μL of 10 mM Tris (pH 8.0) at 60°C. DNA quantity was measured using a Qubit fluorometer (Thermo Fisher Scientific, USA). Samples were gently mixed via inversion throughout extraction to obtain high-molecular-weight DNA.

Library preparation was performed using a Ligation sequencing kit (Oxford Nanopore Technologies, UK). The sample was run on a MinION Mk1B sequencer with an R9.4.1 flow cell. Base calling was performed using Guppy (version 4.0.15+56940742; Oxford Nanopore Technologies, UK). Adapters were removed using Porechop (63) and trimmed for quality (value = 9) and length (>500 bp) using NanoFilt (version 2.7.1 [64]). MinION reads, in combination with previously generated Illumina reads (32), were assembled using Unicycler (normal mode, version 0.4.9b [65]). Illumina reads were mapped back to the assembled closed genome using CLC Genomics Workbench (version 20.0.4; Qiagen, Germany). Annotations were performed using NCBI’s Prokaryotic Genome Annotation Pipeline (PGAP) (version 2020-09-24.build4894 [66]), and genes were classified into broad functional categories based on results from BlastKOALA and KEGG Ontology (67, 68). A perl script was used to calculate GC content and GC skew using a window size of 1,000 and a step size of 100 (69), and sequences were visualized using Circos (version 0.69-8 [70]). Taxonomic analysis and species classification of the closed genome were performed using the Type Strain Genome Server on 16 June 2021 (71). To determine plasmid incompatibility groups, plasmid replicons were searched using CLC Genomics Workbench for palindromic regions that were then aligned to previously described repABC-plasmid incompatibility groups (23, 72). Other closed *Sulfitobacter* genomes were downloaded from NCBI and searched for similar plasmid sequences. Mash distance searches (73) were also performed, using the PLSDB plasmid database (version 2020_06_29 [74]) to identify closely related plasmids in other taxa. Gene synteny and homology were then visualized using Easyfig (version 2.2.5 [75]). Sequence data were submitted to GenBank under the accession numbers CP072613 through CP072617.

### Generation of derivative strains.

*S. pontiacus* CB-D (lysogenized with prophage φ-D) was previously used to generate *S. pontiacus* CB-A (lysogenized with prophage φ-A) via superinfection of CB-D with exogenous temperate phage φ-A, which resulted in prophage substitution in a subset of the host population (32). Here, we leveraged this result to generate derivative strains of CB-A and CB-D using free phage particles generated from induction (Fig. S4), as we lack a phage-cured strain. Derivative φ-A-lysogenized strains were generated via addition of exogenous φ-A (multiplicity of infection [MOI], 0.01) to exponential-phase CB-D cultures (10^7^ CFU/mL). Aliquots were taken at 4 and 8 h postinfection (hpi) and plated for isolation. Derivative φ-D-lysogenized strains were generated via addition of an exogenous mixture of φ-D and φ-A (from a superinfection of CB-D with φ-A; MOI, 0.0005) to exponential-phase CB-A cultures (10^7^ CFU/mL). Aliquots were taken at 8 and 24 hpi and plated for isolation. Subsets of isolates were PCR screened for the presence of phages using phage-specific primers (Table S5) as previously described (32). Isolates were then passaged for isolation three times on agar medium before a second round of PCR screening. Prophage integration was confirmed using PCR primers amplifying the junction between host and phage DNA (Table S5). Thermocycling conditions for these primers were as follows: 3 min at 95°C; then 35 cycles of 40 s at 95°C, 40 s at 57.3°C, and 40 s at 72°C; followed by 5 min at 72°C.

10.1128/msphere.00930-21.5 TABLE S5 Oligonucleotides used in this study Table S5, PDF file, 0.2 MB. Copyright © 2022 Tuttle et al. 2022 Tuttle et al. https://creativecommons.org/licenses/by/4.0/ This content is distributed under the terms of the Creative Commons Attribution 4.0 International license.

### Plasmid copy number enumeration and detection within strains.

DNA was extracted from bacterial strains using a DNeasy blood and tissue kit (Qiagen, Germany) according to the manufacturer’s instructions. Plasmid copy number in the parental strains were enumerated via qPCR using a DNA Engine Opticon 2 system with the Opticon Monitor 3.1.32 software package (Bio-Rad Laboratories, Inc., USA) following established SYBR green-based methods (76). Two or more primer sets for each plasmid were designed to amplify unique regions and were compared to reference primer sets designed to amplify the chromosome (Table S5). Reactions were set up in 25-μL volumes with 12.5-μL TB green premix *Ex Taq* (TaKaRa Bio, Inc., Japan), primers at concentrations optimized for amplification efficiency, and three quantities of DNA template (25 ng, 5 ng, and 2.5 ng). Thermocycling conditions were as follows: 2 min at 95°C; then 40 cycles of 20 s at 95°C, 20 s at 57°C, and 20 s at 72°C; followed by 5 min at 72°C and a melt curve from 50°C to 100°C at 1°C/s. Fluorescence measurements were taken after each cycle and every 1°C of the melt curve. Melt curves consistently showed single peaks, indicating high amplification specificity. Copy number determination was performed using a previously published approach (77). Averages of individual plasmid- and chromosome-specific primer sets were used in calculating the plasmid-chromosome (P-C) ratio for all DNA template concentrations in biological triplicate.

Primers specific to unique regions of plasmids (Table S5) were used to amplify DNA and determine their presence or absence within derivative strains. Thermocycling conditions for contig-specific primers with ∼500-bp products were as follows: 5 min at 95°C; then 30 cycles of 1 min at 95°C, 1 min at 59°C, and 1 min at 72°C; followed by 10 min at 72°C. Thermocycling conditions for plasmid-specific primers used in qPCR were the same as described above.

### Phage enumeration.

Phage abundance from induced cultures was measured via plaque assay and qPCR using phage-specific primers as previously described (32). Free-phage titers of uninduced cultures were measured via a phage dilution assay to detect spontaneous prophage induction (SPI). Overnight cultures of permissible host strains were subcultured in SMM and grown until the early exponential phase (10^7^ CFU/mL). Then, 200-μL aliquots were added to 5 mL top agar (SMM; 0.70% noble agar) and poured onto SMM bottom agar plates. Phages were serially diluted (10^0^ to 10^−5^) in SMM, and 10 μL of each dilution or no-phage control was spotted onto solidified agar in triplicate. Plates were then incubated at room temperature (∼25°C) for 48 h and observed for zones of clearing.

### Biofilm assays.

Relative biofilm formation was quantified via a standard crystal violet biofilm assay as previously described (32). Pairwise Wilcoxon tests were performed to identify significant differences in biofilm formation using the R package ggpubr (version 0.2.5 [78]).

### Transmission electron microscopy.

Overnight cultures grown in SMM were concentrated via centrifugation for 30 min at 4,000 rpm, washed with 10 mM Tris buffer (pH 8.0), and centrifuged again for 2 min at 4,000 rpm prior to negative staining (79). Pelleted cells were placed on ice and fixed for 1 h in a 3% (vol/vol) glutaraldehyde solution. Aliquots (10 μL) were added to a 200-mesh Formvar/carbon-coated copper grid and adsorbed for 3 min at ambient temperature followed by staining for 1 min with 0.5% (wt/vol) aqueous uranyl acetate. Samples were examined on a Zeiss Libra 200 HT FE MC transmission electron microscope using an acceleration voltage of 80 kV.

### Detection of plasmid sequences in *Tara* Oceans data sets.

The biogeographical distribution and abundance of plasmid replicon genes was determined using the Ocean Gene Atlas online server (80). Nucleotide sequences of host and phage genes were searched against the *Tara* Oceans Microbiome Reference Gene Catalog database (version 2; OM-RGCv2) using BLASTn with an expected threshold of 1 × 10^−10^. The abundance of BLAST hits for each sampling station and depth was normalized as a percentage of total reads within the sample for the prokaryotic size fraction (0.22 to 1.6 μm or 0.22 to 3 μm). Assessment of long range homology between plasmid sequences and *Tara* Oceans prokaryotic assemblies was performed using a custom Tara BLAST server (version 2.0.0.beta4, available at http://bioinfo.szn.it/tara-blast-server/ [81]). Nucleotide sequences of whole plasmids were searched against the *Tara* assemblies prokaryotic database (62,500,683 total sequences) using BLASTN 2.9.0+ and the default E value cutoff of 1 × 10^−5^. BLAST hits were searched against the European Nucleotide Archive to determine sampling stations and depths from which sequences were derived.
