# Supplementary material for: Plasmid-Mediated Stabilization of Prophages
Source: mSphere. 2022 Mar 21;7(2):e00930-21. doi: 10.1128/msphere.00930-21 (PMC9044938; doi:10.1128/msphere.00930-21)
Supplement: TABLE S1 [file msphere.00930-21-s0006.pdf]

Table S1. *Sulfitobacter pontiacus* CB-D genome properties.

**Genomic element**	**Length (bp)**	**Protein-coding genes**	**tRNAs**	**rRNA operons**	**tmRNAs**	**ncRNAs**	**GC content (%)**	**Accession**	**Regions of new sequence in the closed CB-D genome**	
									**Genes**	**Length (bp)**
Chromosome	3281733	3168	47	3	1	2	60.5%	CP072613	IS3 family transposase<br>IS3 family transposase<br>rRNA operon<br>rRNA operon<br>rRNA operon<br>EF-Tu<br>EF-Tu	1388<br>1388<br>6272<br>6278<br>6209<br>1034<br>1034
pSpoCB-1	176823	168	0	0	0	0	60.3%	CP072614	--	--
pSpoCB-2	132295	148	0	0	0	0	59.7%	CP072615	--	--
pSpoCB-3	130875	122	0	0	0	0	55.1%	CP072616	--	--
pSpoCB-4	72015	66	0	0	0	0	57.6%	CP072617	IS256 family transposase<br>IS256 family transposase	1174<br>1174
**Whole genome**	**3793741**	**3672**	**47**	**3**	**1**	**2**	**60.3%**	**--**		
