# Supplementary material for: Plasmid-Mediated Stabilization of Prophages
Source: mSphere. 2022 Mar 21;7(2):e00930-21. doi: 10.1128/msphere.00930-21 (PMC9044938; doi:10.1128/msphere.00930-21)
Supplement: TABLE S2 [file msphere.00930-21-s0007.pdf]

Table S2. Properties of complete *Sulfitobacter* genomes.

Strain	Genome size (bp)	Number of plasmids	Percent extrachromosomal	Number of prophages[α]	Accession
Sulfitobacter pontiacus CB-D	3793741	4	13.5%	1	CP072613
Sulfitobacter alexandrii AM1-D1	4691010	5	18.1%	1	CP018076
Sulfitobacter pontiacus S1704	3551353	3	14.9%	0	CP049344
Sulfitobacter pseudonitzschiae H46	4933696	9	21.1%	0	CP054599
Sulfitobacter pseudonitzschiae SMR1	5121602	7	30.2%	0	CP022415
Sulfitobacter sp. B30-2	2928325	0	0.0%	1	CP065429
Sulfitobacter sp. BSw21498	3244919	1	4.5%	0	CP040753
Sulfitobacter sp. D7	3963108	5	14.9%	0	CP020694
Sulfitobacter sp. JL08	4323690	0	0.0%	0	CP025815
Sulfitobacter sp. SK011	4190786	0	0.0%	0	CP025803
Sulfitobacter sp. SK012	5176795	3	7.5%	0	CP025804
Sulfitobacter sp. SK025	3946853	6	23.1%	2	CP025808
Sulfitobacter sp. THAF37	4264270	7	19.1%	0	CP045372
**AVERAGE**	**4163858**	**4**	**13.9%**	**--**	**--**

[α] Detected by PHASTER (Arndt et al., 2016).

Arndt D, Grant JR, Marcu A, Sajed T, Pon A, Liang Y, Wishart DS. 2016. PHASTER: a better, faster version of the PHAST phage search tool. Nucleic Acids Research 44:W16-W21.
