# Supplementary figures and images for: Plasmid-Mediated Stabilization of Prophages
Source: mSphere. 2022 Mar 21;7(2):e00930-21. doi: 10.1128/msphere.00930-21 (PMC9044938; doi:10.1128/msphere.00930-21)

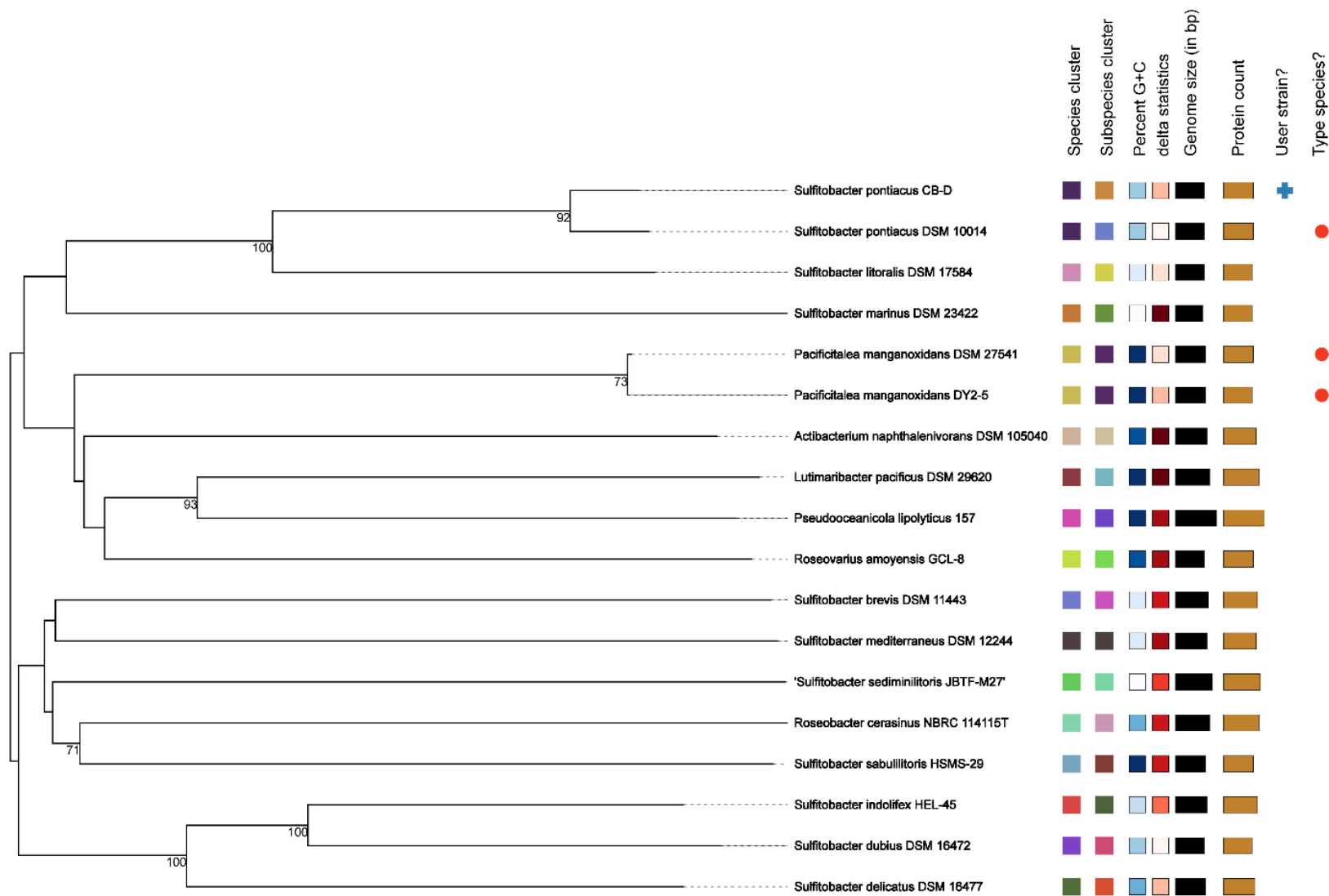

Supplement: FIG S1 [file msphere.00930-21-s0001.pdf]

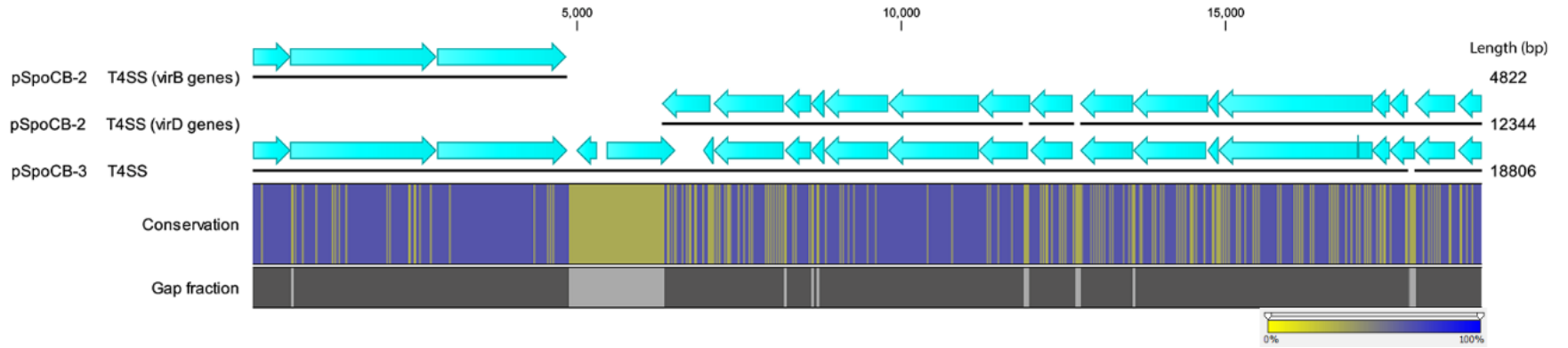

Supplement: FIG S2 [file msphere.00930-21-s0002.pdf]

CB-D

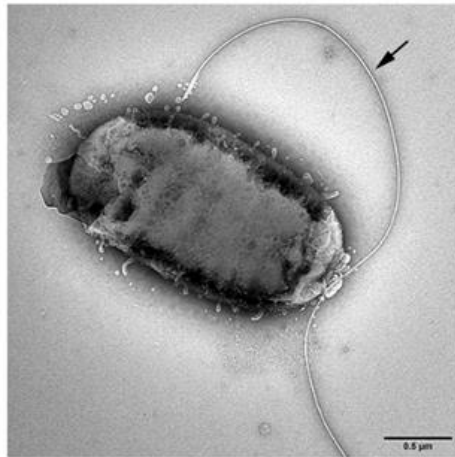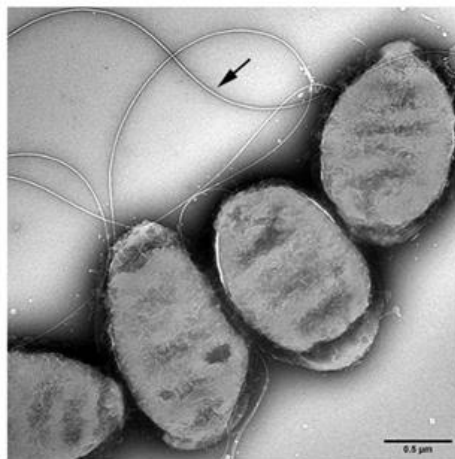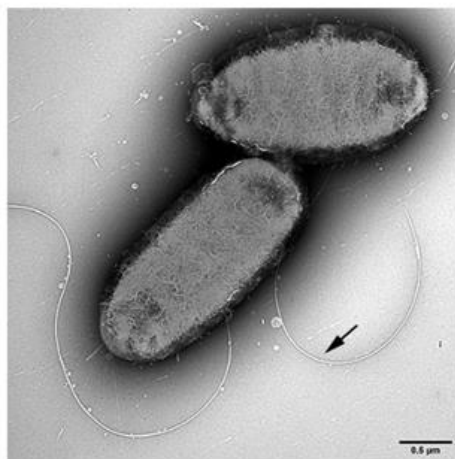

CB-A

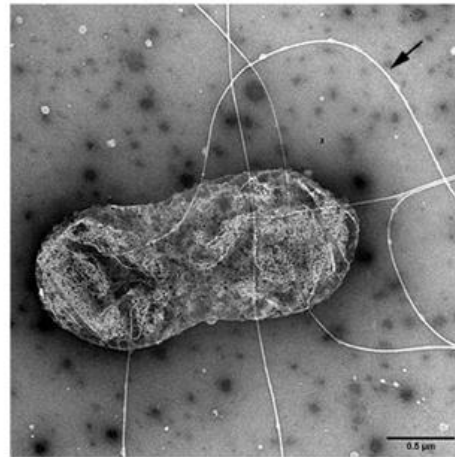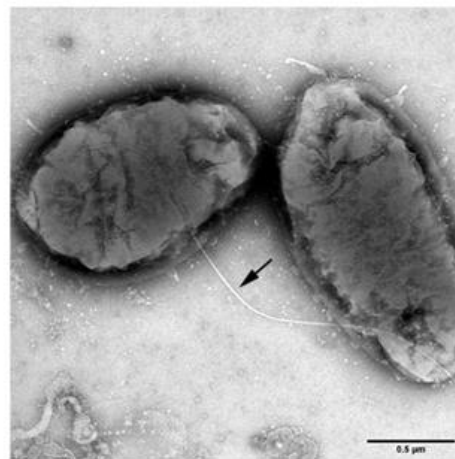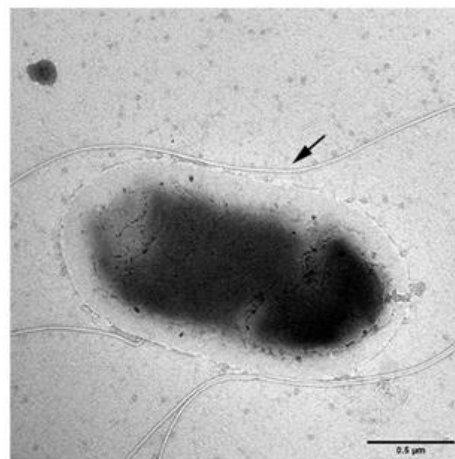

Supplement: FIG S3 [file msphere.00930-21-s0003.pdf]

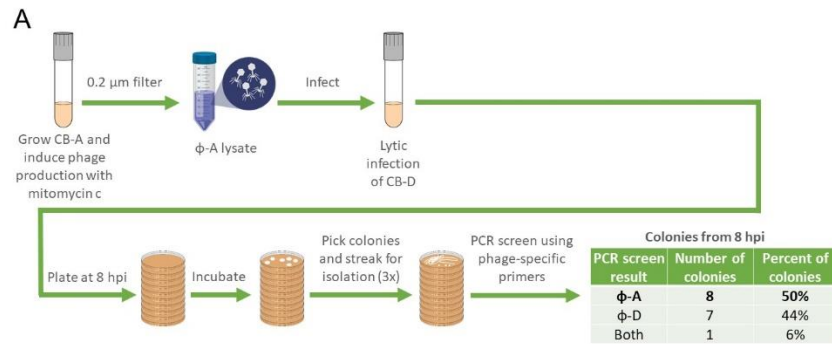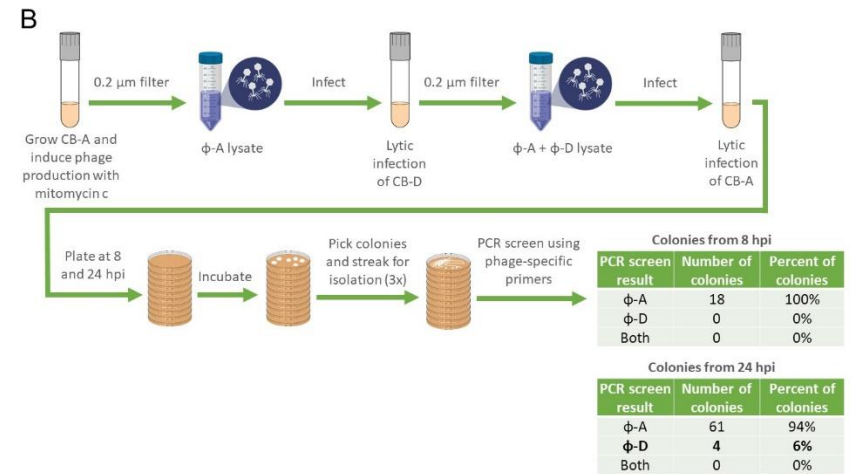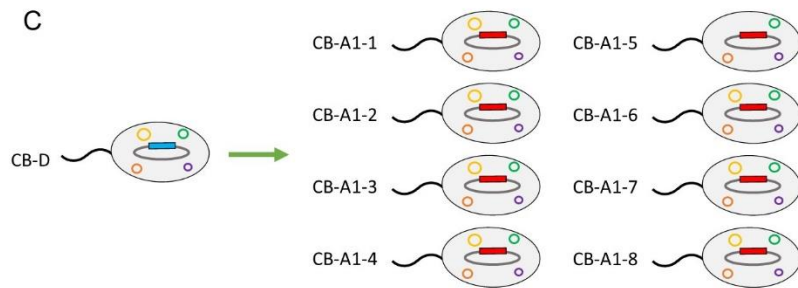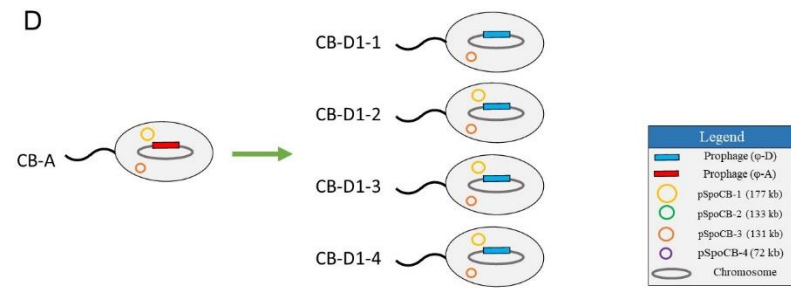

E

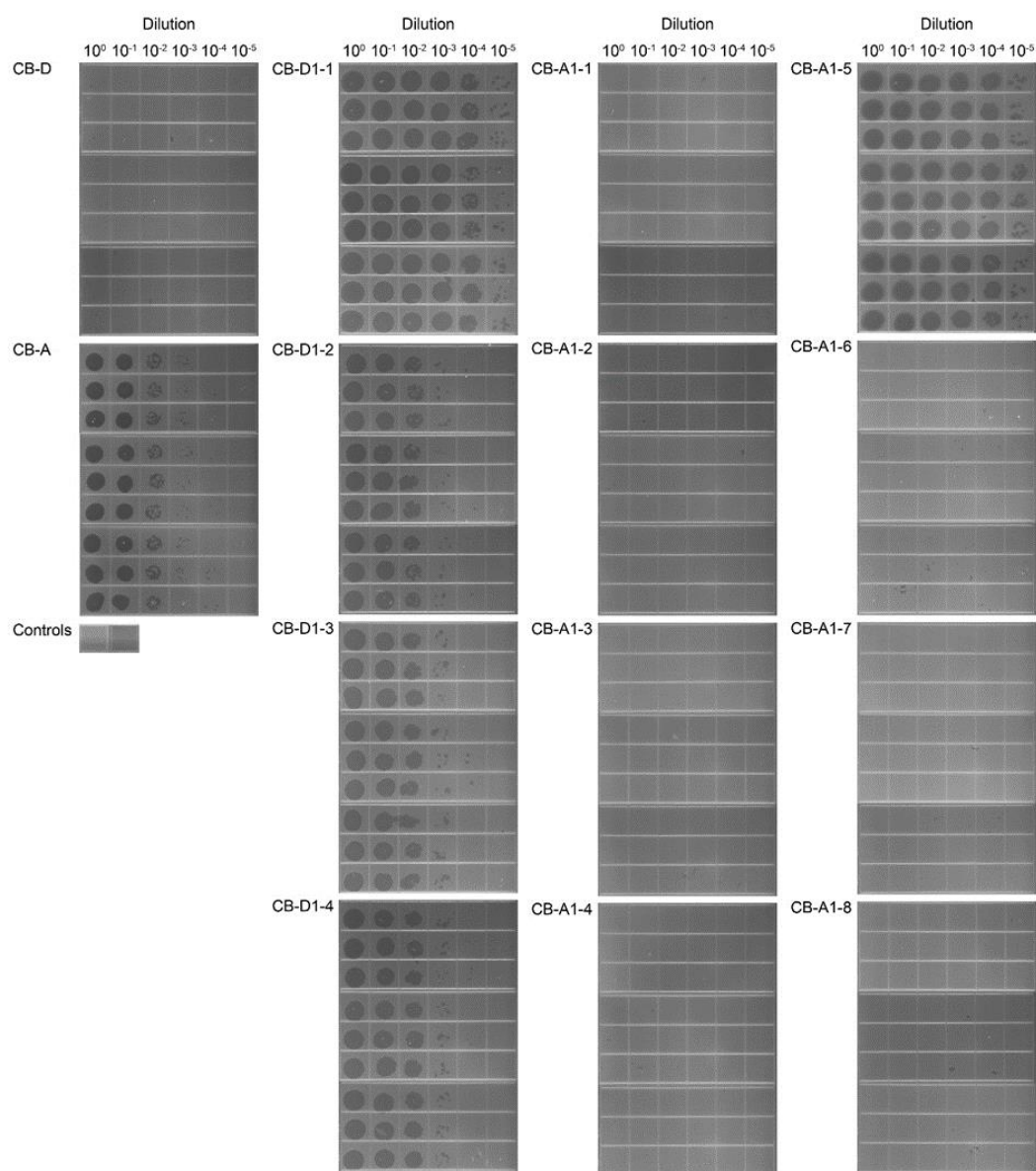

Supplement: FIG S4 [file msphere.00930-21-s0004.pdf]

A

pSpoCB-1

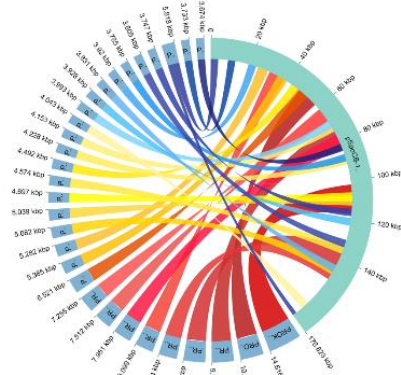

B

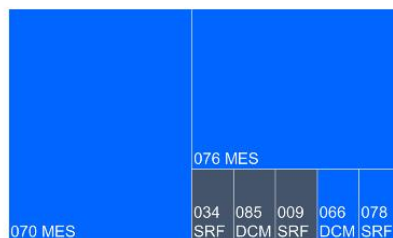

pSpoCB-2

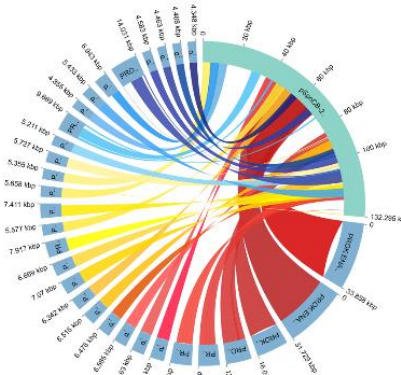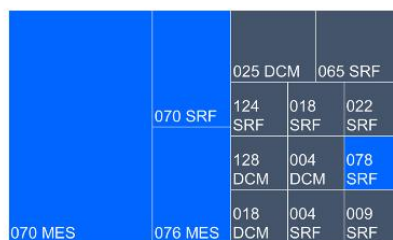

pSpoCB-3

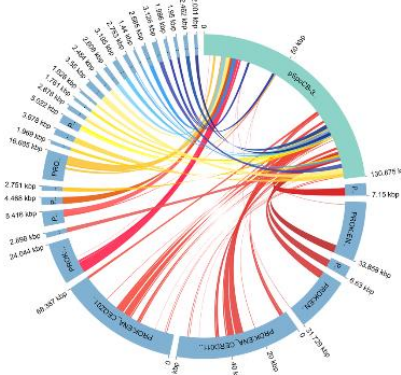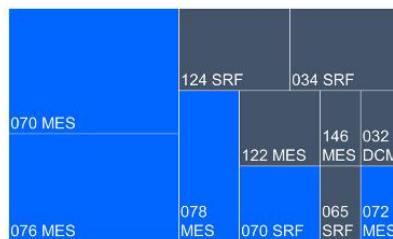

pSpoCB-4

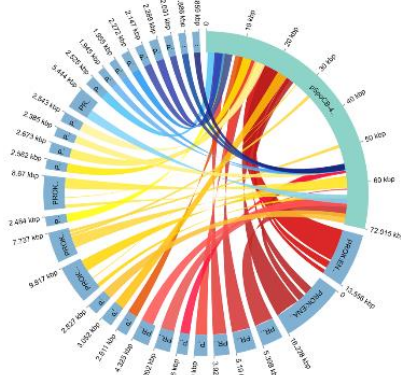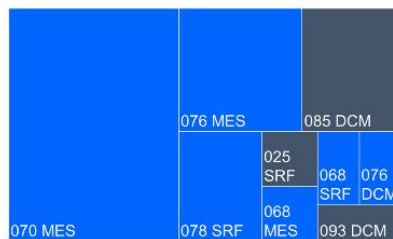

Supplement: FIG S5 [file msphere.00930-21-s0005.pdf]
