# Supplementary material for: Plasmid-Mediated Stabilization of Prophages
Source: mSphere. 2022 Mar 21;7(2):e00930-21. doi: 10.1128/msphere.00930-21 (PMC9044938; doi:10.1128/msphere.00930-21)
Supplement: TABLE S3 [file msphere.00930-21-s0008.pdf]

Table S3. Sequencing coverage statistics of CB-D and CB-A Illumina reads from Basso et al. (2020) mapped to the complete CB-D genome.

Genomic element	Number of reads mapped		Coverage (mean ± standard deviation)		Total length of zero coverage regions	
	**CB-D**	**CB-A**	**CB-D**	**CB-A**	**CB-D**	**CB-A**
Chromosome	6195770	7123125	284.4 ± 46.32	326.83 ± 54.38	0	8546[β]
pSpoCB-1	297959	35	253.49 ± 19.76	0.03 ± 0.18	0	171809
pSpoCB-2	214769	273	244.29 ± 18.45	0.31 ± 0.57	0	97811
pSpoCB-3	210210	235358	241.8 ± 19.15	270.52 ± 20.64	0	0
pSpoCB-4	96783	8	202.16 ± 17.72	0.02 ± 0.14	0	70930
**Whole genome**	**7015491**	**7358799**	**278.11 ± 46.34**	**321.66 ± 62.55**[α]	**0**	**349,096**

[α] Excludes no coverage regions for CB-A genome

[β] Representative of regions where the prophages ϕA and ϕD differ

Basso JTR, Ankrah NYD, Tuttle MJ, Grossman AS, Sandaa R-A, Buchan A. 2020. Genetically similar temperate phages form coalitions with their shared host that lead to niche-specific fitness effects. The ISME Journal doi:10.1038/s41396-020-0637-z.
