# Supplementary material for: Plasmid-Mediated Stabilization of Prophages
Source: mSphere. 2022 Mar 21;7(2):e00930-21. doi: 10.1128/msphere.00930-21 (PMC9044938; doi:10.1128/msphere.00930-21)
Supplement: TABLE S4 [file msphere.00930-21-s0009.pdf]

Table S4. List of strains and bacteriophages used in this study.

**Strain or bacteriophage name**			**Description**	**Source**
Strains	*Sulfitobacter pontiacus*	CB-D	Also known as strain CB2047, isolated from an *Emiliania huxleyi* phytoplankton bloom in Raunefjorden, Norway	Ankrah et al. (2014a)
		CB-A	Generated from superinfection of CB-D with ϕ-A	Basso et al. (2020)
		CB-A1-1	Derivative of CB-D from superinfection with ϕ-A	This study
		CB-A1-2	Derivative of CB-D from superinfection with ϕ-A	This study
		CB-A1-3	Derivative of CB-D from superinfection with ϕ-A	This study
		CB-A1-4	Derivative of CB-D from superinfection with ϕ-A	This study
		CB-A1-5	Derivative of CB-D from superinfection with ϕ-A	This study
		CB-A1-6	Derivative of CB-D from superinfection with ϕ-A	This study
		CB-A1-7	Derivative of CB-D from superinfection with ϕ-A	This study
		CB-A1-8	Derivative of CB-D from superinfection with ϕ-A	This study
		CB-D1-1	Derivative of CB-A from superinfection with ϕ-D	This study
		CB-D1-2	Derivative of CB-A from superinfection with ϕ-D	This study
		CB-D1-3	Derivative of CB-A from superinfection with ϕ-D	This study
		CB-D1-4	Derivative of CB-A from superinfection with ϕ-D	This study
Bacteriophages	*Podoviridae*	ϕ-A	Also known as ϕCB2047A, isolated from an *Emiliania huxleyi* phytoplankton bloom in Raunefjorden, Norway	Ankrah et al. (2014b)
	Podoviridae	ϕ-D	Isolated as a prophage within *Sulfitobacter pontiacus* CB-D	Ankrah et al. (2014a)

Ankrah NY, Lane T, Budinoff CR, Hadden MK, Buchan A. 2014a. Draft Genome Sequence of Sulfitobacter sp. CB2047, a Member of the Roseobacter Clade of Marine Bacteria, Isolated from an Emiliania huxleyi Bloom. Genome Announcements 2.

Ankrah NY, Budinoff CR, Wilson WH, Wilhelm SW, Buchan A. 2014b. Genome Sequences of Two Temperate Phages, PhiCB2047-A and PhiCB2047-C, Infecting Sulfitobacter sp. Strain 2047. Genome Announcements 2.

Basso JTR, Ankrah NYD, Tuttle MJ, Grossman AS, Sandaa R-A, Buchan A. 2020. Genetically similar temperate phages form coalitions with their shared host that lead to niche-specific fitness effects. The ISME Journal doi:10.1038/s41396-020-0637-z.
