# Supplementary material for: Plasmid-Mediated Stabilization of Prophages
Source: mSphere. 2022 Mar 21;7(2):e00930-21. doi: 10.1128/msphere.00930-21 (PMC9044938; doi:10.1128/msphere.00930-21)
Supplement: TABLE S5 [file msphere.00930-21-s0010.pdf]

Table S5. Oligonucleotides used in this study.

Primer name	Sequence (5' to 3')	Gene target	Primer concentration$^{\alpha}$	Product length	Purpose/ experiment	Reference
2047PP1.for	TATTCATAGCGAGGCGCAGT	endolysin	10 µM	128 bp	ϕ-D presence and enumeration	Basso et al. (2020)
2047PP1.rev	ATACCTGCCCAACGTCACAG	endolysin	10 µM			
2047A-C.for	CCCATGTGTATGTCGCCTCT	endolysin	10 µM	110 bp	ϕ-A presence and enumeration	Basso et al. (2020)
2047A-C.rev	CAGCGTTGAAAAAGGCTCTG	endolysin	10 µM			
alaS_577for	GGAGAGAGTGACGCATGGAT	alanine tRNA hydrolase	500 nM	143 bp	Chromosome reference for plasmid enumeration	Basso et al. (2020)
alaS_719rev	CAGCTGTCGAGATGGACGTA	alanine tRNA hydrolase	500 nM			
map_164for	TCACGCAGATGATCGAAGAC	methionine aminopeptidase	500 nM	171 bp	Chromosome reference for plasmid enumeration	Basso et al. (2020)
map_334rev	CATCGACAATTACGGTGACG	methionine aminopeptidase	500 nM			
jxn761U_for	GGCCAGCATAACCGTTTCC	histidine kinase	10 µM	662 bp	Phage integration site confirmation	Basso et al. (2020)
jxn761U_rev	GCACATCGATAGGGTCACG	hypothetical protein	10 µM			
jxn1105D_for	GGGAGGCATGAGCGTAGAA	hypothetical protein	10 µM	485 bp	Phage integration site confirmation	Basso et al. (2020)
jxn1105D_rev	TGTCGCCAACACCTCTACC	HTH transcriptional regulator	10 µM			
CB-D_contig3_fwd	CGCGCCAGATCATTGAGAAA	glucose-1-phosphate thymidylyltransferase	10 µM	512 bp	pSpoCB-4 presence/absence	This study
CB-D_contig3_rev	AAAGGAGAGGCACGACATCA	intergenic region	10 µM			

CB-D_contig4_fwd	CACGTACCGGAAGCAATGAG	hypothetical protein	10 µM	513 bp	pSpoCB-4 presence/absence	This study
CB-D_contig4_rev	GGAGATTGCGCTAAACCCAG	intergenic region	10 µM			
CB-D_contig6_fwd	GGAAAGAAGCTGTCGAGGTC	cytochrome C550	10 µM	500 bp	pSpoCB-2 presence/absence	This study
CB-D_contig6_rev	TCCGATTGTAAGCGTCTCTG	cytochrome C550	10 µM			
CB-D_contig7-1_fwd	CATGGAGCAGGAACACATCG	multidrug transporter	10 µM	511 bp	pSpoCB-1 presence/absence	This study
CB-D_contig7-1_rev	CTGTTTGATGACCTCGAGCG	multidrug transporter	10 µM			
CB-D_contig7-2_fwd	GATCAACACCAAACGCATTC	ABC transporter permease	10 µM	518 bp	pSpoCB-1 presence/absence	This study
CB-D_contig7-2_rev	TGCCCCATCAGCATATTAAA	intergenic region	10 µM			
CB-D_contig7-3_fwd	GCGAATACCCAGACCTTCAT	flagellar hook protein	10 µM	519 bp	pSpoCB-1 presence/absence	This study
CB-D_contig7-3_rev	GACGACCTCTTGTCCGGTAT	flagellar hook protein	10 µM			
CB-D_contig7-4_fwd	GGCTTTGGATTGGTCTTTGT	flagellar biosynthesis protein	10 µM	512 bp	pSpoCB-1 presence/absence	This study
CB-D_contig7-4_rev	CCTAAAGCCCATGATCCACT	flagellar biosynthesis protein	10 µM			
CB-D_contig12_fwd	CAGACCGTAACGACCCAACT	intergenic region	10 µM	508 bp	pSpoCB-3 presence/absence	This study
CB-D_contig12_rev	TTTAAGGTTGGTCCGACGAC	hypothetical protein	10 µM			
contig_2-1_qPCR_F	GCTCTGCCGCTTTACGATAC	hypothetical protein	1000 nM	142 bp	Chromosome reference for plasmid enumeration	This study
contig_2-1_qPCR_R	ACCGCGTTCGACTATTTCAC	hypothetical protein	500 nM			

contig_2-2_qPCR_F	AGGCCATATCAAGCGTGAAC	hydantoin utilization protein A	1000 nM	159 bp	Chromosome reference for plasmid enumeration	This study
contig_2-2_qPCR_R	AGGATTTCGTGCAAACCATC	hydantoin utilization protein A	1000 nM			
contig_3-1_qPCR_F	TATCAGGGCCGTTAGGTACG	UDP-glucuronate 5-epimerase	1000 nM	151 bp	pSpoCB-4 presence and enumeration	This study
contig_3-1_qPCR_R	ATGGCCCTGTTCAAGTTCAC	UDP-glucuronate 5-epimerase	1000 nM			
contig_3-2_qPCR_F	ATGAGCAGAAAACGGATTGG	glycosyl transferase	1000 nM	148 bp	pSpoCB-4 presence and enumeration	This study
contig_3-2_qPCR_R	TCCAAAACCCCGATGATAAG	glycosyl transferase	1500 nM			
contig_4-1_qPCR_F	ACCCATTTCTTCGCATGTTC	intergenic region	1000 nM	147 bp	pSpoCB-4 presence and enumeration	This study
contig_4-1_qPCR_R	GGCACCCAAAACCATGATAC	intergenic region	1000 nM			
contig_4-2_qPCR_F	AGCACCATATCGAAGGATCG	nodulation protein NoeE	500 nM	157 bp	pSpoCB-4 presence and enumeration	This study
contig_4-2_qPCR_R	GACAGGGGAAAGAGTTGCTG	nodulation protein NoeE	1500 nM			
contig_6-1_qPCR_F	TAATCGAGCCAACCCGATAG	hypothetical protein	1500 nM	150 bp	pSpoCB-2 presence and enumeration	This study
contig_6-1_qPCR_R	CCCATTGACTGACACACCAG	hypothetical protein	1500 nM			
contig_6-2_qPCR_F	CAGTCAGCAGCTCGAAAGTG	copper resistance protein	500 nM	143 bp	pSpoCB-2 presence and enumeration	This study
contig_6-2_qPCR_R	CGGTGCTGAAGCCTCTTATC	copper resistance protein	1000 nM			
contig_7-1_qPCR_F	AGGGCTGAACATGACAATCC	glycosyl transferase family 1	500 nM	160 bp	pSpoCB-1 presence and enumeration	This study
contig_7-1_qPCR_R	GTCTGAGGCTTCCGTCATTC	glycosyl transferase family 1	1500 nM			

contig_7-2_qPCR_F	TCGACGATCTCATGAACCAG	flagellar motor switch protein FliG	1500 nM	148 bp	pSpoCB-1 presence and enumeration	This study
contig_7-2_qPCR_R	ATCTGGCGGATCTCATCAAC	flagellar motor switch protein FliG	1000 nM			
contig_9-1_qPCR_F	ACGCCATTGGATTGAAAGAC	intergenic region	1000 nM	148 bp	Chromosome reference for plasmid enumeration	This study
contig_9-1_qPCR_R	TTTGCTATCACAGCGGACAC	intergenic region	1500 nM			
contig_9-2_qPCR_F	TCGAGGTACTGGTCCTCCTG	C4-dicarboxylate ABC transporter permease	1000 nM	143 bp	Chromosome reference for plasmid enumeration	This study
contig_9-2_qPCR_R	GCAATGGCTTCGAGTTTCTC	3-keto-5-aminohexanoate cleavage protein	500 nM			
contig_12-1_qPCR_F	TGGCTTGGGTCGAGTAATTC	hypothetical protein	1000 nM	143 bp	pSpoCB-3 presence and enumeration	This study
contig_12-1_qPCR_R	CAAGAAGGGATACGCTCTGC	hypothetical protein	1500 nM			
contig_12-2_qPCR_F	ATGAAGGGCATCAGGTTGAC	ABC transporter ATP-binding protein	1500 nM	158 bp	pSpoCB-3 presence and enumeration	This study
contig_12-2_qPCR_R	ACCCTGTTCGATGGACTGAC	ABC transporter ATP-binding protein	1000 nM			

[α] For primers used to enumerate plasmid copy numbers relative to chromosome copies, all permutations of 1500 nM, 1000 nM, 500 nM, and 100 nM forward and reverse primer combinations were tested for optimal qPCR efficiency.

Basso JTR, Ankrah NYD, Tuttle MJ, Grossman AS, Sandaa R-A, Buchan A. 2020. Genetically similar temperate phages form coalitions with their shared host that lead to niche-specific fitness effects. The ISME Journal doi:10.1038/s41396-020-0637-z.
